# Supplementary material for: Ligand recognition mechanism of the human relaxin family peptide receptor 4 (RXFP4)
Source: Nat Commun. 2023 Jan 30;14:492. doi: 10.1038/s41467-023-36182-z (PMC9886975; doi:10.1038/s41467-023-36182-z)
Supplement: Supplementary file 1 — Supplementary Information [file 41467_2023_36182_MOESM1_ESM.pdf]

Supplementary Information

Ligand recognition mechanism of the human relaxin family peptide receptor 4 (RXFP4)

Brief description of what this file includes:

Supplementary Fig. 1 | Characterization of recombinant INSL5.

Supplementary Fig. 2 | Synthesis and characterization of DC591053.

Supplementary Fig. 3 | Functional validations of the receptor constructs and purification of the complexes.

Supplementary Fig. 4 | Cryo-EM data processing and validation.

Supplementary Fig. 5 | Near-atomic resolution model of the complexes in the cryo-EM density maps.

Supplementary Fig. 6 | Conformational changes upon RXFP4 activation.

Supplementary Fig. 7 | Comparison of the peptide-binding pocket of RXFP4 with other class A GPCRs.

Supplementary Fig. 8 | Molecular dynamics (MD) simulations of INSL5-bound active RXFP4.

Supplementary Fig. 9 | MD simulations of INSL5 and its B chain.

Supplementary Fig. 10 | Peptidomimetic agonism and key residues on receptor subtype selectivity.

Supplementary Fig. 11 | Superimposition of INSL5 from the INSL5–RXFP4–G_i complex structure with insulin or IGF-1.

Supplementary Fig. 12 | Superimposition of INSL5 to insulin or IGF-1 in complex with cognate receptors.

Supplementary Fig. 13 | Gating strategy of the cell surface expression assay.

Supplementary Table 1 | Cryo-EM data collection, refinement and validation statistics.

Supplementary Table 2 | Interactions of INSL5, compound 4 and DC591053 with RXFP4.

Supplementary Table 3 | Ligands-mediated inhibition of forskolin-induced cAMP accumulation.

Supplementary Table 4 | Cell surface expression and effects of residues mutation on ligands-mediated inhibition of forskolin-induced cAMP accumulation.

Supplementary Table 5 | Effects of key residue mutation on receptor subtype selectivity.

Supplementary Table 6 | Primers used in this study, related to Figures 2 and 3, Supplementary Figures 3 and 10 and Supplementary Tables 3, 4 and 5.

Supplementary Table 7 | Details of restraints applied during MD simulations.

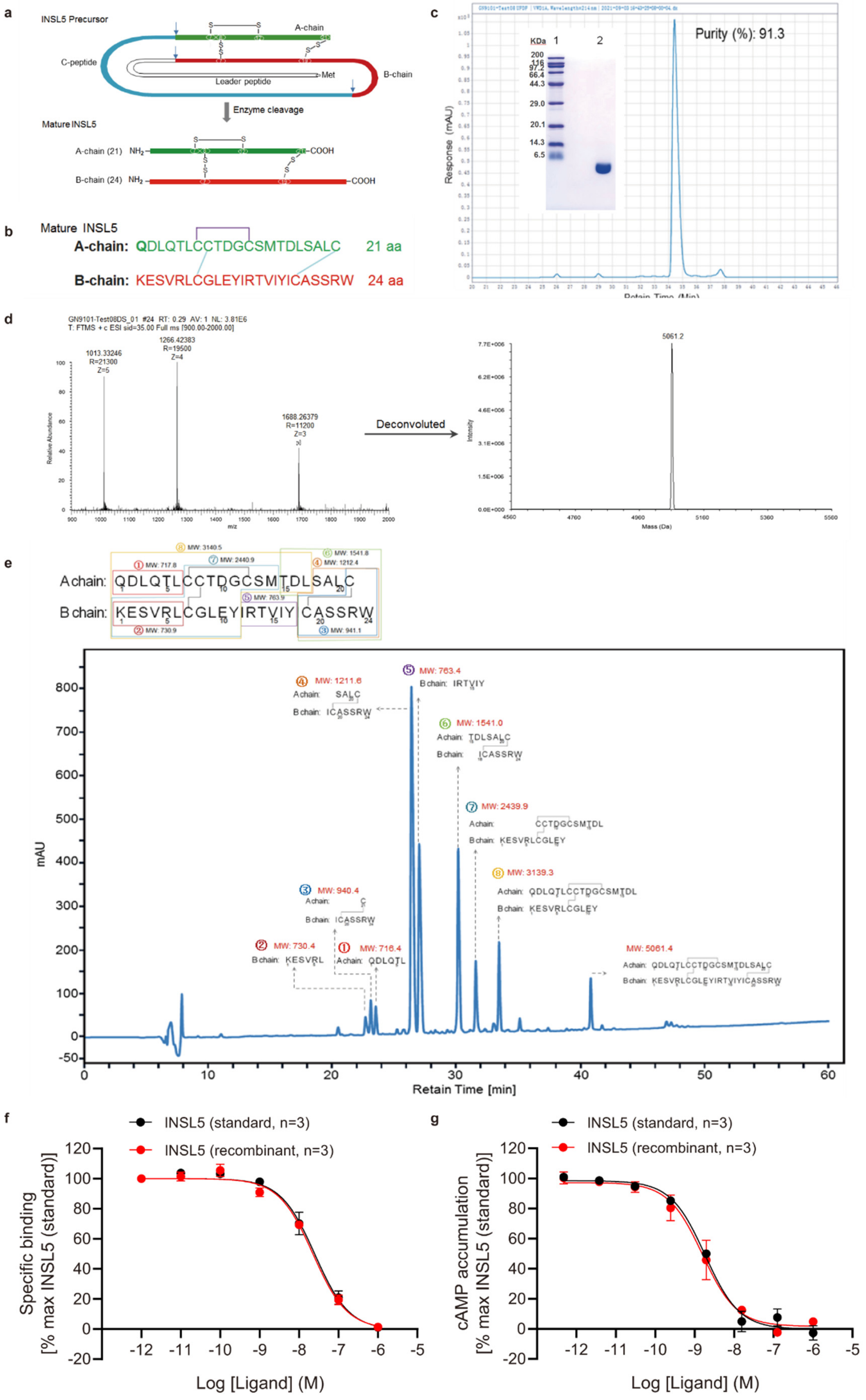
a
INSL5 Precursor
A-chain
C-peptide
Leader peptide
Met
B-chain
Enzyme cleavage
Mature INSL5
A-chain (21) NH2
COOH
B-chain (24) NH2
COOH
b
Mature INSL5
A-chain: QDLQTLCCTDGCSMTDLSALC 21 aa
B-chain: KESVRLCGLEYIRTVIYICASSRW 24 aa
c
Purity (%): 91.3
KDa 1 2
200
116
97.2
66.4
44.3
29.0
20.1
14.3
6.5
Response (mAU)
d
Relative Abundance
1013.33246
R=21300
Z=5
1266.42383
R=19500
Z=4
1688.26379
R=11200
Z=3
m/z
Deconvoluted
5061.2
Intensity
Mass (Da)
e
A chain: QDLQTLCCTDGCSMTDLSALC
B chain: KESVRLCGLEYIRTVIYICASSRW
MW: 717.8
MW: 3140.5
MW: 2440.9
MW: 1541.8
MW: 1212.4
MW: 763.9
MW: 730.9
MW: 941.1
mAU
MW: 1211.6
A chain: SALC
B chain: ICASSRW
MW: 763.4
B chain: IRTVIY
MW: 1541.0
A chain: TDLSALC
B chain: ICASSRW
MW: 2439.9
A chain: CCTDGCSMTDL
B chain: KESVRLCGLEY
MW: 3139.3
A chain: QDLQTLCCTDGCSMTDL
B chain: KESVRLCGLEY
MW: 940.4
A chain: C
B chain: ICASSRW
MW: 730.4
B chain: KESVRL
MW: 716.4
A chain: QDLQTL
MW: 5061.4
A chain: QDLQTLCCTDGCSMTDLSALC
B chain: KESVRLCGLEYIRTVIYICASSRW
Retain Time [min]
f
INSL5 (standard, n=3)
INSL5 (recombinant, n=3)
Specific binding
[% max INSL5 (standard)]
Log [Ligand] (M)
g
INSL5 (standard, n=3)
INSL5 (recombinant, n=3)
cAMP accumulation
[% max INSL5 (standard)]
Log [Ligand] (M)

Supplementary Figure 1. Characterization of recombinant INSL5. a, The structure of the INSL5 precursor and its conversion to mature INSL5. **b,** Amino acid sequence of the recombinant INSL5 used in this study. The dash indicates disulfide bond. **c,** Purity analysis of recombinant INSL5 by non-reducing SDS-PAGE and (reverse phase high-performance liquid chromatography) RP-HPLC. Well 1 is the marker, well 2 is a typical batch of recombinant INSL5. These experiments were repeated independently three times with similar results. The purity of INSL5 in this batch is 91.3%. **d,** Mass spectrometry (MS) analysis of INSL5. The measured molecular mass of 5,061.2 Da corresponds to the expected value (5,062.9 Da) of the INSL5 (N-terminal Q of A chain not converted to pE). **e,** Chymotrypsin-generated peptide mapping by liquid chromatograph (LC)-LC/MS and assignments for the amino acid sequences of the peptides. Measured molecular mass in red; structure assignment and theoretical molecular weights in black. **f-g,** The recombinant INSL5 was able to bind and activate RXFP4 as determined by europium-labeled ligand competitive binding **(f)** and cAMP accumulation assays **(g)** compared to INSL5 (standard), a control peptide containing native amino acids (N-terminal Q of A chain converted to pE). Data shown are means ± S.E.M. of three independent experiments labeled in the parentheses. Source data are provided as a Source Data file.

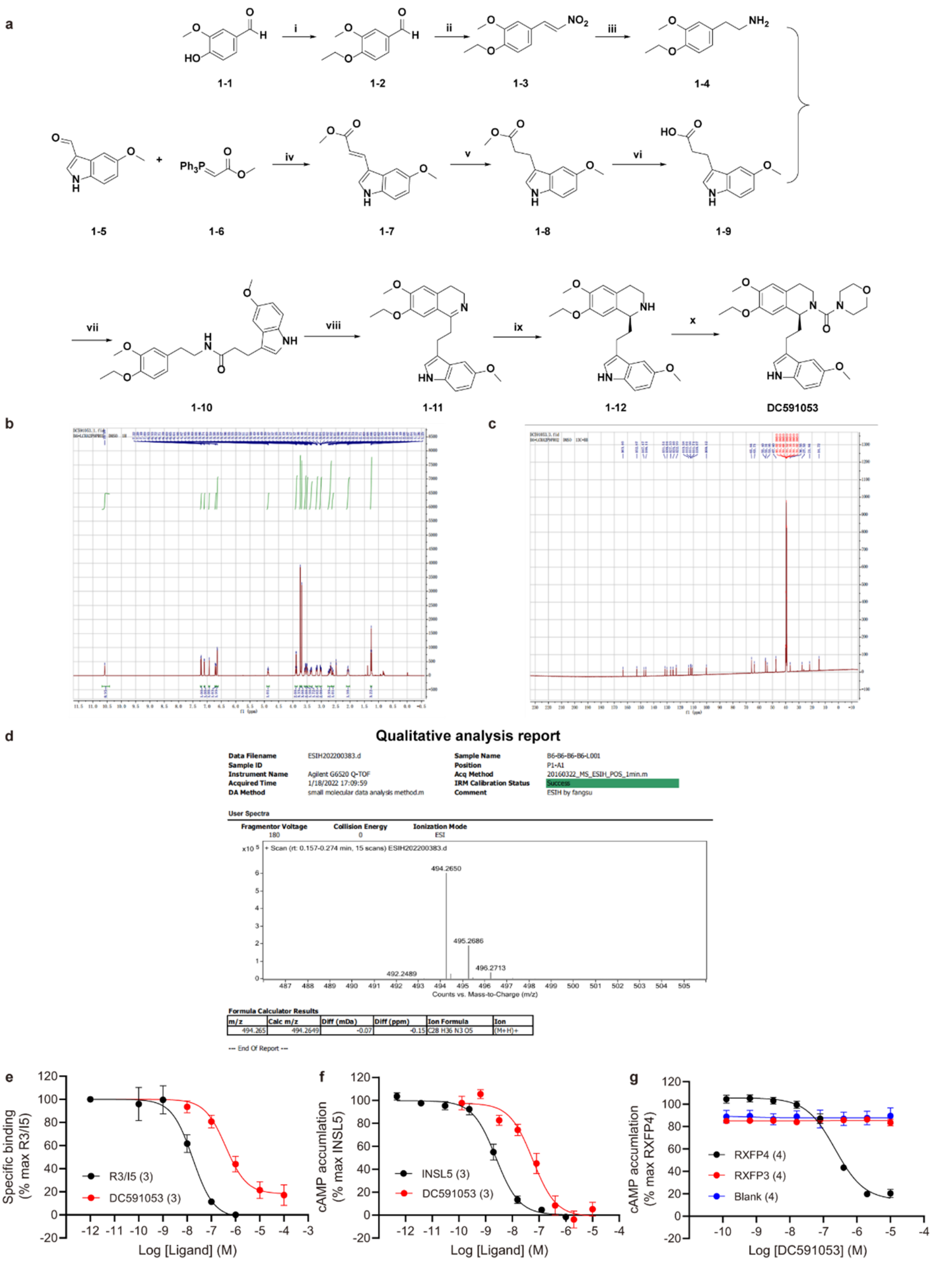

Supplementary Figure 2. Synthesis and characterization of DC591053. a, Synthetic route to the compound DC591053. (i) CH_3CH_2I, CH_3CN, K_2CO_3, 80 °C, Ar, 90%; (ii) CH_3COONH_4, CH_3NO_2, 100 °C ,1 h, 65%; (iii) $LiAlH_4$, anhydrous THF, 0 - rt, Ar, 78%; (iv) Toluene, Ar, 110 °C, 16 h, 60%; (v) 10% $Pd(OH)_2$/C, H_2, MeOH, 40 °C, 5 h, 95%; (vi) 1M NaOH, MeOH, rt, 93%; (vii) HATU, TEA, rt, DCM, 90%; (viii) $POCl_3$, CH_3CN, 80 °C, Ar, 92%; (ix) RuCl[(*R,R*)-Tsdpen](*p*-cymene), $AgSbF_6$, $La(OTf)_3$, HCOONa, H_2O/MeOH= 1:1, Ar, rt, 60%; (x) 4-morpholinecarbonyl chloride,

DIPEA, DCM, 0 °C to rt, 90%. **b,** ^{1}H NMR spectrum of compound DC591053 (500 MHz, DMSO-d_6). **c,** ^{13}C NMR spectrum of compound DC591053 (125 MHz, DMSO-d_6). **d,** HRMS spectrum of compound DC591053**. e-f,** DC591053 was able to bind and activate RXFP4 as determined by europium-labeled ligand competitive binding **(e)** and cAMP accumulation assays **(f).** Data shown are means ± S.E.M. of three independent experiments labeled in the parentheses. **g,** DC591053 neither cross-reacted with RXFP3 nor showed any activity in parental cells. Data shown are means ± S.E.M. of four independent experiments labeled in the parentheses. Source data are provided as a Source Data file.

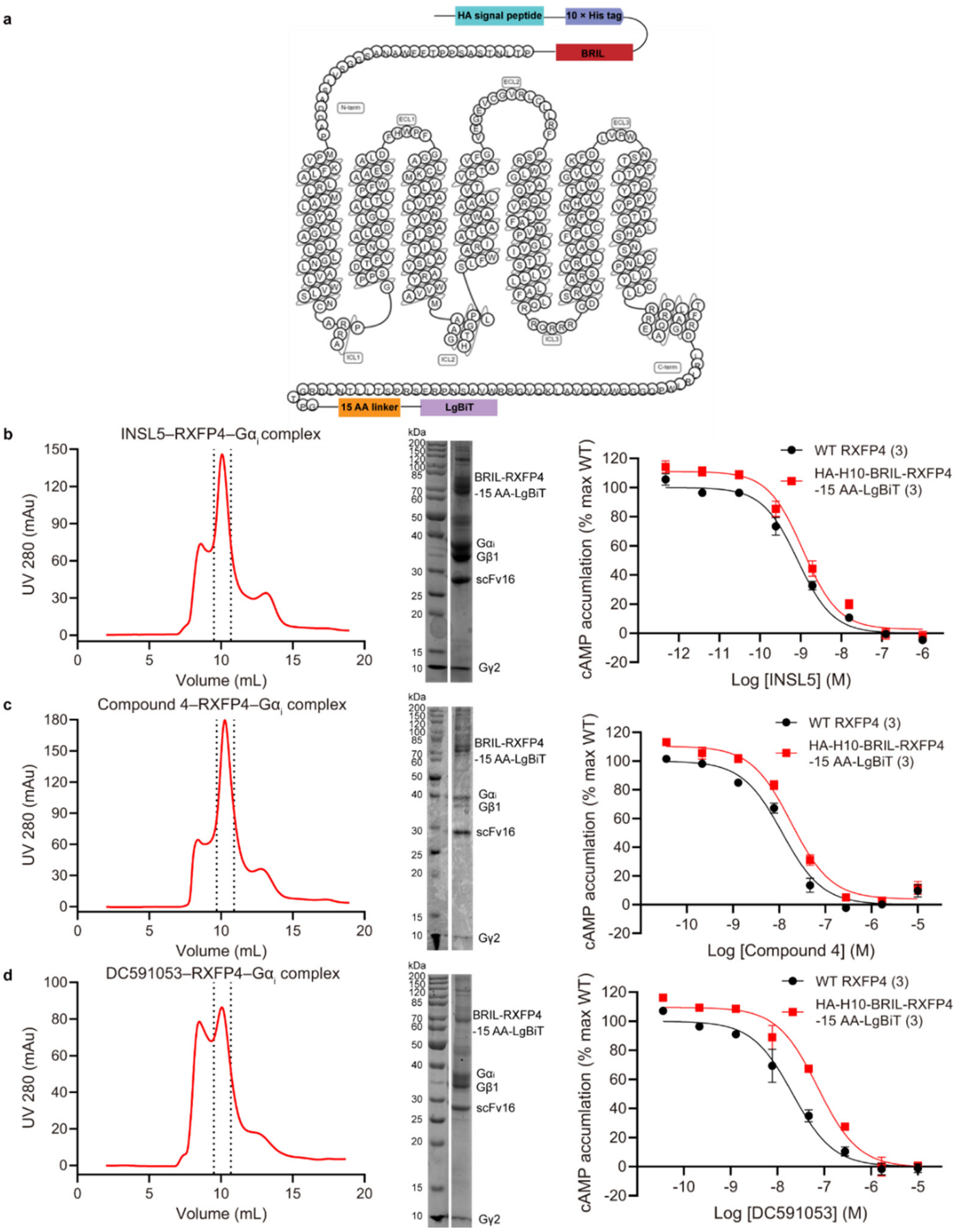

Supplementary Figure 3. Functional validations of the receptor constructs and purification of the complexes. a, Schematic diagram of the receptor constructs used for structure determination. **b-d,** Analytical size-exclusion chromatography (left) and SDS-PAGE/Coomassie blue stain (middle) of the purified INSL5–RXFP4–G_i **(b)**, Compound 4–RXFP4–G_i **(c)** and DC591053–RXFP4–G_i **(d)**. These experiments were repeated independently three times with similar results. The right panel in **b-d** is INSL5, compound 4 and DC591053 induced cAMP accumulation in wild-type (WT) and modified RXFP4 constructs. Data shown were means ± S.E.M. from three independent experiments shown in the parentheses. Supplementary Tables 3 provides detailed statistical evaluation such as *P* values and numbers of independent experiments (*n*). Source data are provided as a Source Data file.

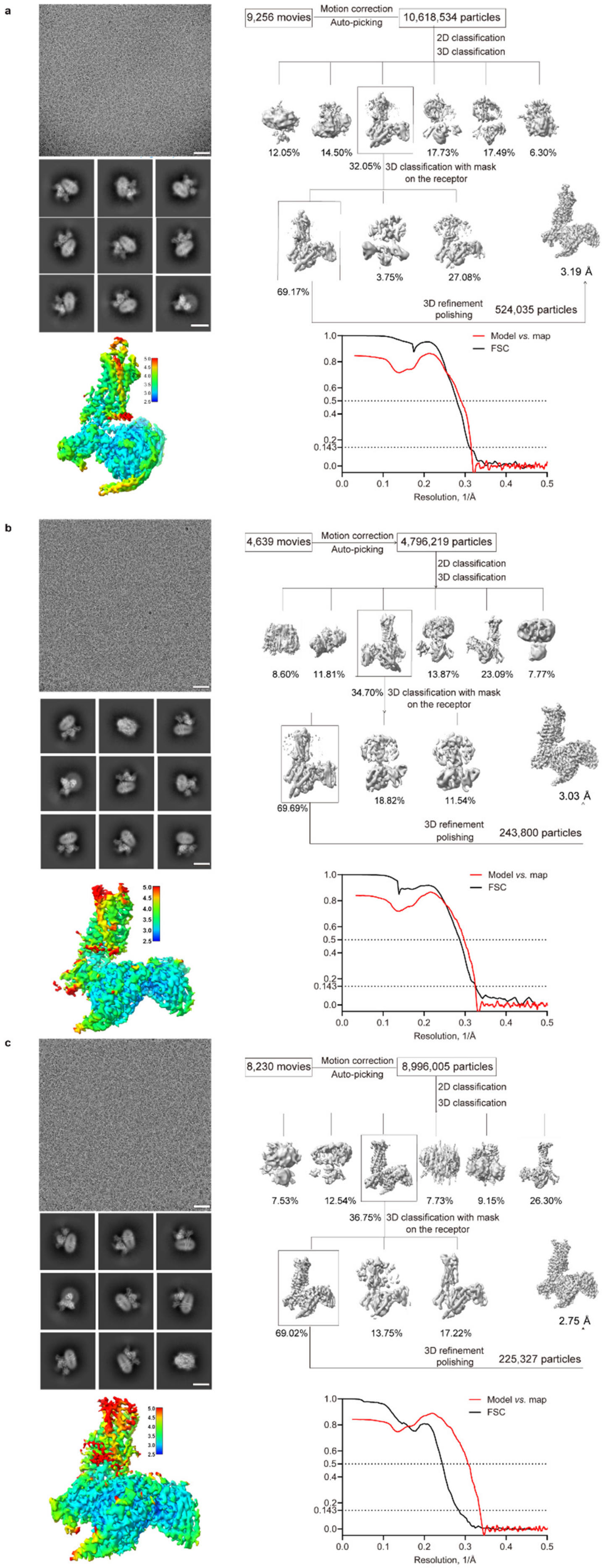

a
9,256 movies
Motion correction
Auto-picking
10,618,534 particles
2D classification
3D classification
12.05%
14.50%
17.73%
17.49%
6.30%
32.05%
3D classification with mask on the receptor
3.75%
27.08%
69.17%
3.19 Å
3D refinement polishing
524,035 particles
Model vs. map
FSC
Resolution, 1/Å
b
4,639 movies
Motion correction
Auto-picking
4,796,219 particles
2D classification
3D classification
8.60%
11.81%
13.87%
23.09%
7.77%
34.70%
3D classification with mask on the receptor
18.82%
11.54%
69.69%
3.03 Å
3D refinement polishing
243,800 particles
Model vs. map
FSC
Resolution, 1/Å
c
8,230 movies
Motion correction
Auto-picking
8,996,005 particles
2D classification
3D classification
7.53%
12.54%
7.73%
9.15%
26.30%
36.75%
3D classification with mask on the receptor
69.02%
13.75%
17.22%
2.75 Å
3D refinement polishing
225,327 particles
Model vs. map
FSC
Resolution, 1/Å

Supplementary Figure 4. Cryo-EM data processing and validation. a, INSL5–RXFP4–G_i complex: top left, representative cryo-EM micrograph (scale bar: 40 nm) and two-dimensional (2D) class averages showing distinct secondary structure features from different views (scale bar: 5 nm); top right, flow chart of cryo-EM data processing; bottom left, local resolution distribution map of the complex; bottom right, Fourier shell correlation (FSC) curves of overall refined receptor. **b,** Compound 4–RXFP4–G_i complex: top left, representative cryo-EM micrograph (scale bar: 40 nm) and 2D class averages showing distinct secondary structure features from different views (scale bar: 5 nm); top right, flow chart of cryo-EM data processing; bottom left, local resolution distribution map of the complex; bottom right, FSC curves of overall refined receptor. **c,** DC591053–RXFP4–G_i complex top left, representative cryo-EM micrograph (scale bar: 40 nm) and 2D class averages showing distinct secondary structure features from different views (scale bar: 5 nm); top right, flow chart of cryo-EM data processing; bottom left, local resolution distribution map of the complex; bottom right, FSC curves of overall refined receptor. These experiments were repeated twice independently with similar results. Source data are provided as a Source Data file.

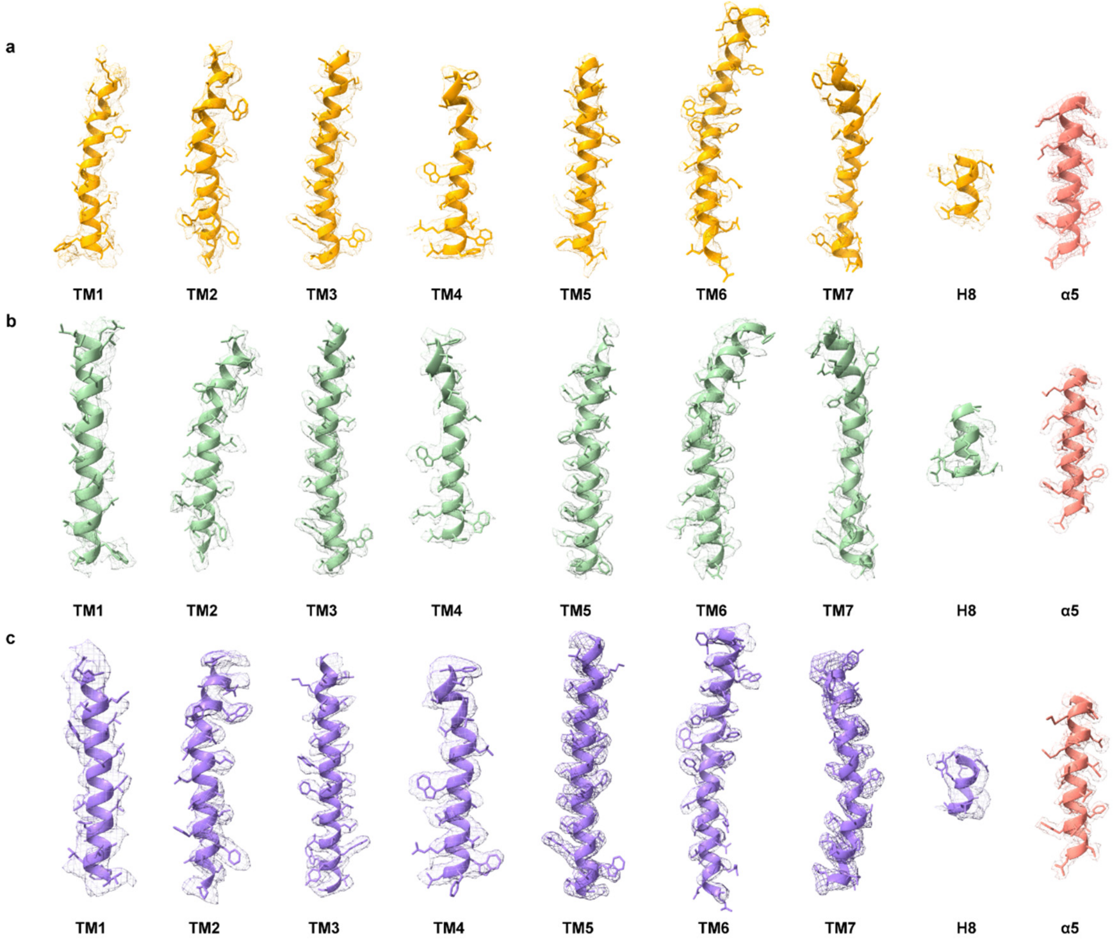

Supplementary Figure 5. Near-atomic resolution model of the complexes in the cryo-EM density maps. a, EM density map and model of the INSL5–RXFP4–G_i complex are shown for all seven-transmembrane (7TM) α-helices, helix 8 and the α5-helix of the G_i subunit. The receptor and α5-helix of the G_i subunit are colored by orange and salmon, respectively. **b,** EM density map and model of the compound 4–RXFP4–G_i complex are shown for all 7TM α-helices, helix 8 and the α5-helix of the G_i subunit. The receptor and α5-helix of the G_i subunit are colored by dark sea green and salmon, respectively. **c,** EM density map and model of the DC591053–RXFP4–G_i complex are shown for all 7TM α-helices, helix 8 and the α5-helix of the G_i subunit. The receptor and α5-helix of the G_i subunit are colored by medium purple and salmon, respectively.

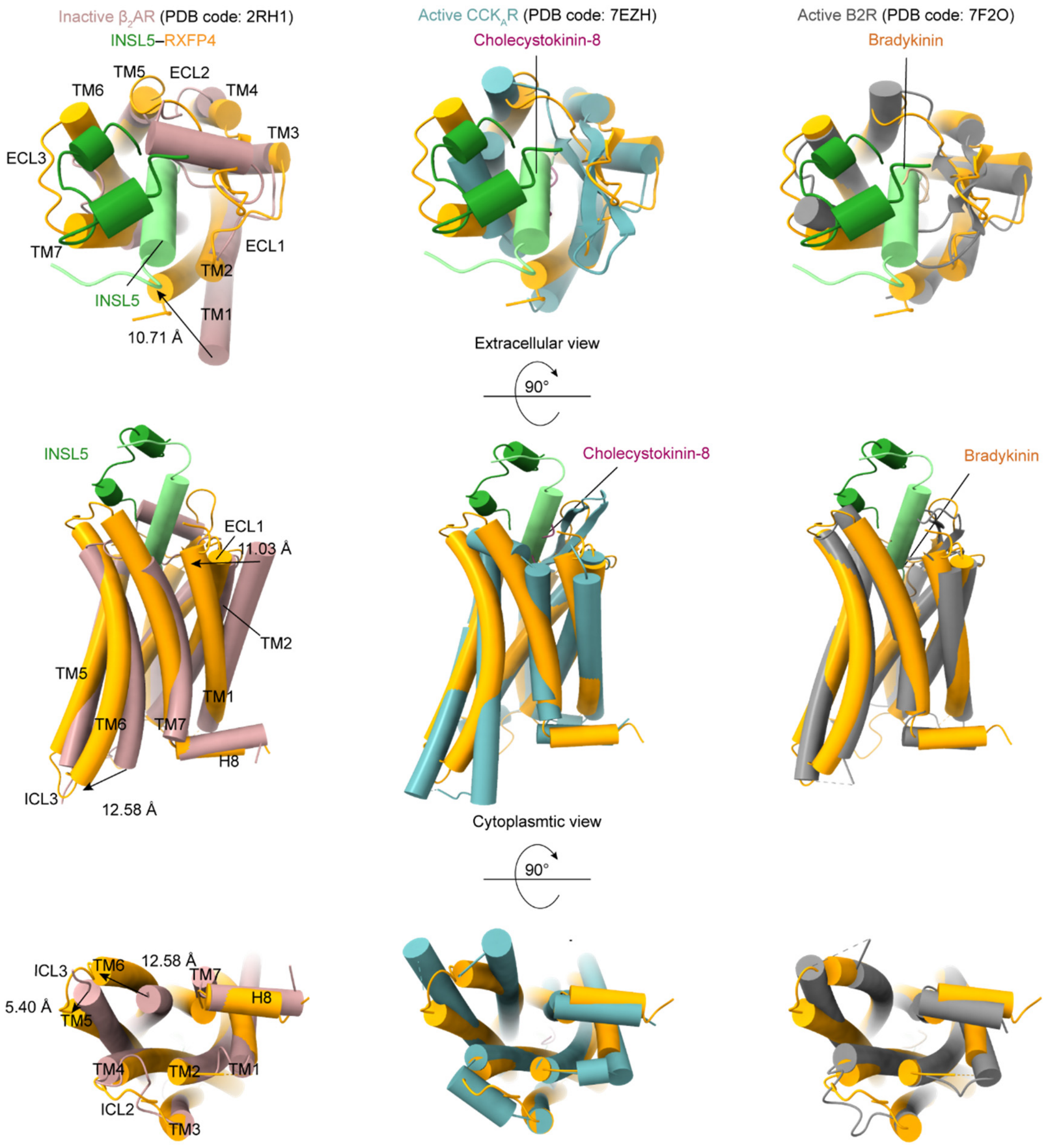

Supplementary Figure 6. Conformational changes upon RXFP4 activation. Comparison of active RXFP4 (orange) with inactive β2-adrenergic receptor (β2AR) (rosy brown, PDB code: 2RH1)[1] and both agonist-bound and G protein-coupled active cholecystokinin A receptor (CCK_AR) (cadet blue, PDB code: 7EZH)[2] and type 2 bradykinin receptor (B2R) (gray, PDB code: 7F2O)[3]. G proteins were omitted for clarity.

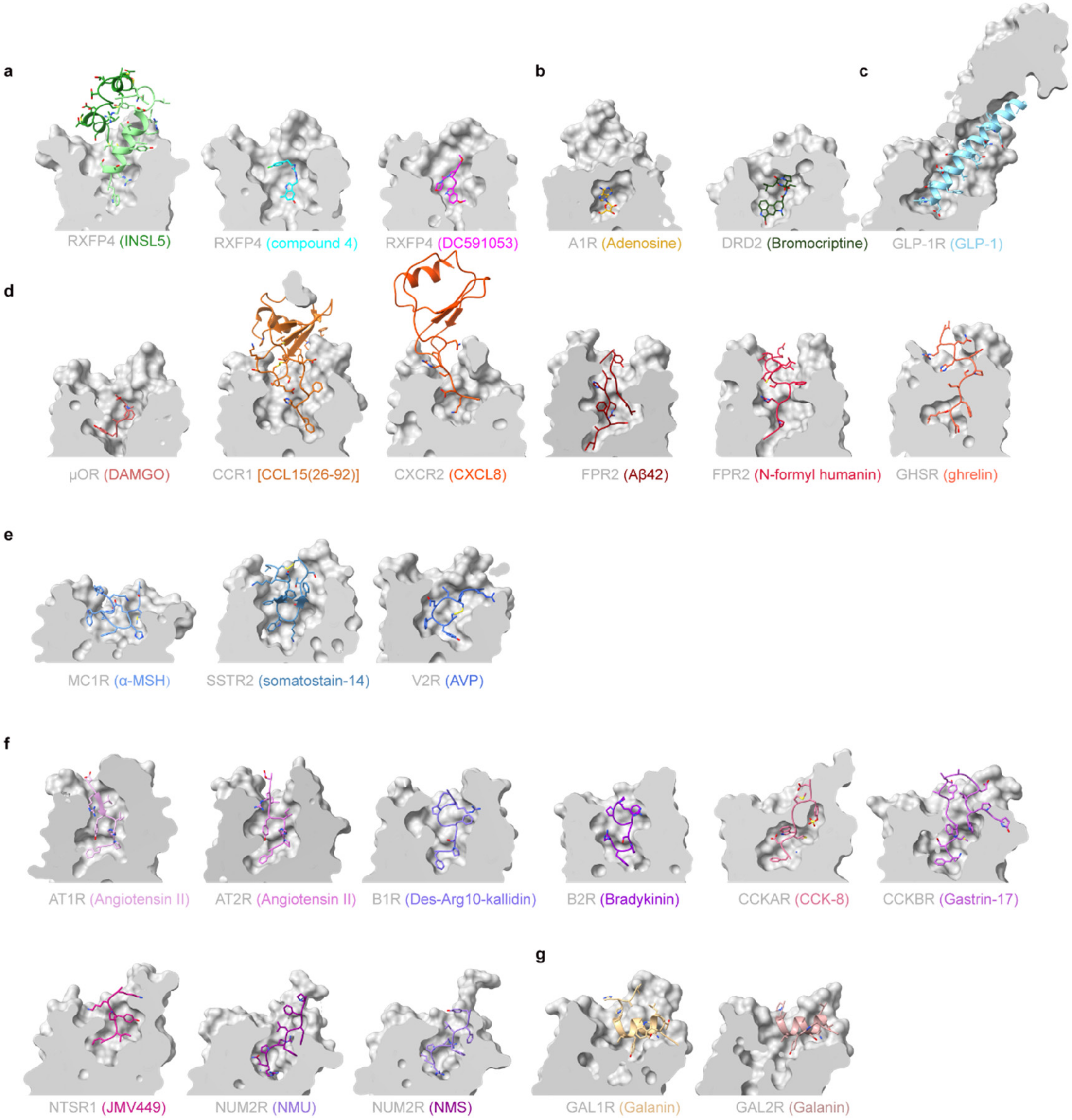

Supplementary Figure 7. Comparison of the peptide-binding pocket of RXFP4 with other class A GPCRs. The binding cavity of INSL5, compound 4 and DC591053-bound RXFP4 (**a**) were compared with that of other GPCRs **(b-g)** including small molecule bound receptors [adenosine in adenosine A_1 receptor (A_1R) (PDB code: 7LD4) and bromocriptine in D2 dopamine receptors (DRD2) (PDB code: 7JVR)] **(b)**, glucagon-like peptide-1 (GLP-1)-bound glucagon-like peptide-1 receptor (GLP-1R) (PDB code: 6X18) **(c)**, the N terminus of peptides inserts into the TMD core such as DAMGO-bound μ-opioid receptor (μOR) (PDB code: 6DDE), C-C chemokine ligand 15 [CCL15(26-92)]-bound C-C chemokine receptor type 1 (CCR1) (PDB code: 7VL9), C-X-C motif chemokine ligand 8 (CXCL8)-bound C-X-C chemokine receptor type 2 (CXCR2) (PDB code: 6LFO), $A\beta_{42}$-bound formyl peptide receptor 2 (FPR2) (PDB code: 7WVY), *N*-formyl humanin-bound FPR2 (PDB code: 7WVX) and ghrelin-bound growth hormone secretagogue receptor (GHSR) (PDB code: 7NA7) **(d)**, the middle segment of cyclic peptides inserts into the TMD binding cavity

such as α-melanocyte-stimulating hormone (α-MSH)-bound melanocortin 1 receptor (MC1R) (PDB code: 7F4D), somatostain-14-bound somatostatin receptor 2 (SSTR2) (PDB code: 7T10) and arginine-vasopressin (AVP)-bound vasopressin receptor 2 (V2R) (PDB code: 7DW9) **(e)**, the C terminus of peptides inserts into the TMD binding pocket such as angiotensin II-bound angiotensin II receptor type 1 (AT1R) (PDB code: 6OS0) and angiotensin II receptor type 2 (AT2R) (PDB code: 6JOD), Des-Arg10-kallidin bound (type 1 bradykinin receptor) B1R (PDB code: 7EIB), bradykinin-bound B2R (PDB code: 7F2O), cholecystokinin-8 (CCK-8)-bound CCK_AR (PDB code: 7EZH) and gastrin-17 bound cholecystokinin B receptor (CCK_BR) (PDB code: 7F8V), JMV449-bound neurotensin receptor 1 (NTSR1) (PDB code: 6OS9), neuromedin U-bound neuromedin U receptor 2 (NUM2R) (PDB code: 7W55) and neuromedin S-bound NUM2R (PDB code: 7W57) **(f)**, and unique binding mode of galanin with galanin receptor 1 (GAL1R) (PDB code: 7WQ3) and galanin receptor 2 (GAL2R) (PDB code: 7WQ4) **(g).**

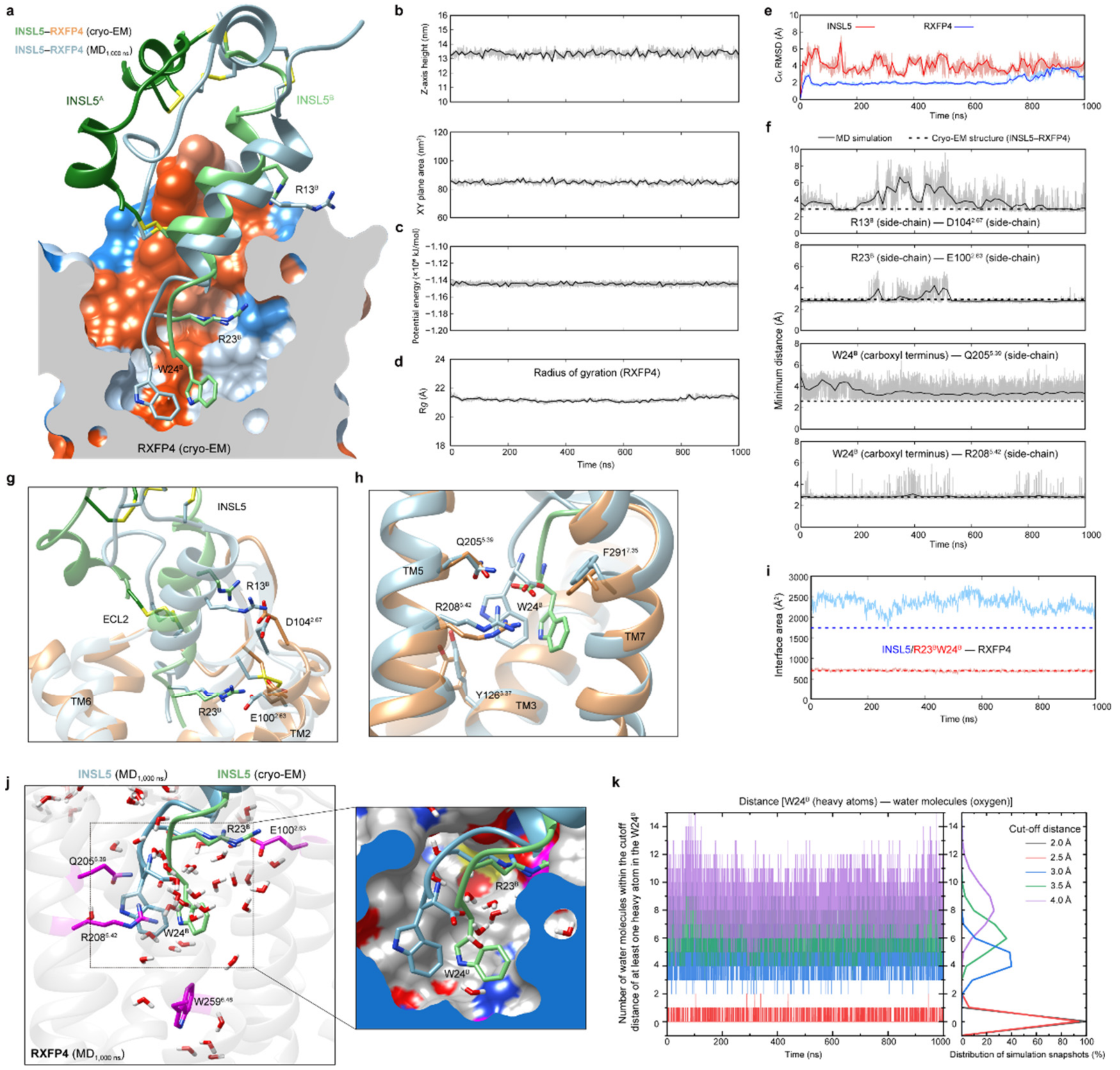

Supplementary Figure 8. Molecular dynamics (MD) simulations of INSL5-bound active RXFP4. a, Comparison of the INSL5 conformation between the final simulation snapshot at 1,000 ns and the cryo-EM structure of INSL5–RXFP4–G_i complex. The key residues in the peptide-receptor interface are shown in sticks. **b,** Time evolution of the Z-axis height (top) and XY plane area (bottom) of the simulation box during MD simulation. **c,** Potential energy fluctuation during MD simulation. **d,** Radius of gyration (R*g*) of RXFP4 during MD simulation. **e,** Root mean squared deviation (RMSD) of Cα positions of the RXFP4 and INSL5, where all snapshots were superimposed on the cryo-EM structure of RXFP4 and INSL5 using the Cα atoms, respectively. **f-h,** Close-up views of the interactions between the C terminal α-helix of INSL5 B chain and receptor residues (**g** and **h**) and their minimum distances during MD simulations (**f**). **i,** The interface area

between RXFP4 and INSL5 (blue) or the two C terminus residues $R23^B$ and $W24^B$ (red), calculated by FreeSASA 2.0. The thick and thin traces represent moving averages and original, unsmoothed values obtained from one single MD simulation trajectory, respectively. **j,** The distribution of water molecules in the orthosteric pocket that overlap with the position of indole group of $W24^B$ in the cryo-EM structure model (green). A close-up view of the internal water molecules distributed around the C terminus of the INSL5 B chain was shown on the right. **k,** Time evolution of the number of water molecules within the cut-off distance of $W24^B$ during MD simulation. Only these water molecules whose oxygen atoms located within the cut-off distance of at least one heavy atom in the $W24^B$ were counted. Five cut-off distances (2.0 Å, 2.5 Å, 3.0 Å, 3.5 Å, and 4.0 Å) were adopted. The MD simulations were repeated independently three times with similar results.

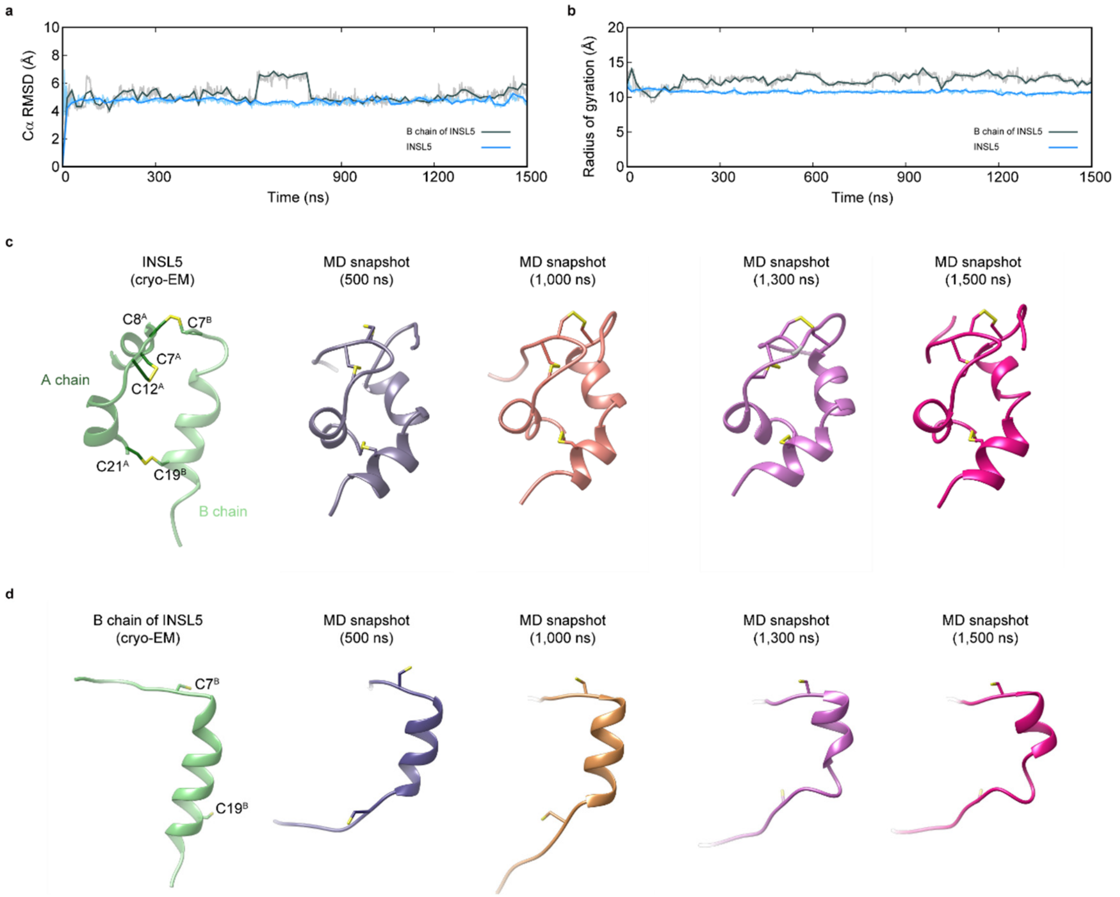

Supplementary Figure 9. MD simulations of INSL5 and its B chain. a, RMSD of Cα positions of the INSL5 and its B chain, where all snapshots were superimposed on the cryo-EM structure of INSL5 and its B chain using the Cα atoms, respectively. **b,** Radius of gyration of non-hydrogen atoms of the INSL5 and its B chain during MD simulations. **c,** Representative snapshots of MD simulations of INSL5. The cysteines are shown in sticks. **d,** Representative snapshots of MD simulations of the B chain of INSL5. The cysteines are shown in sticks. The thick and thin traces represent moving averages and original, unsmoothed values obtained from one single MD simulation trajectory, respectively. The MD simulations were repeated independently three times with similar results.

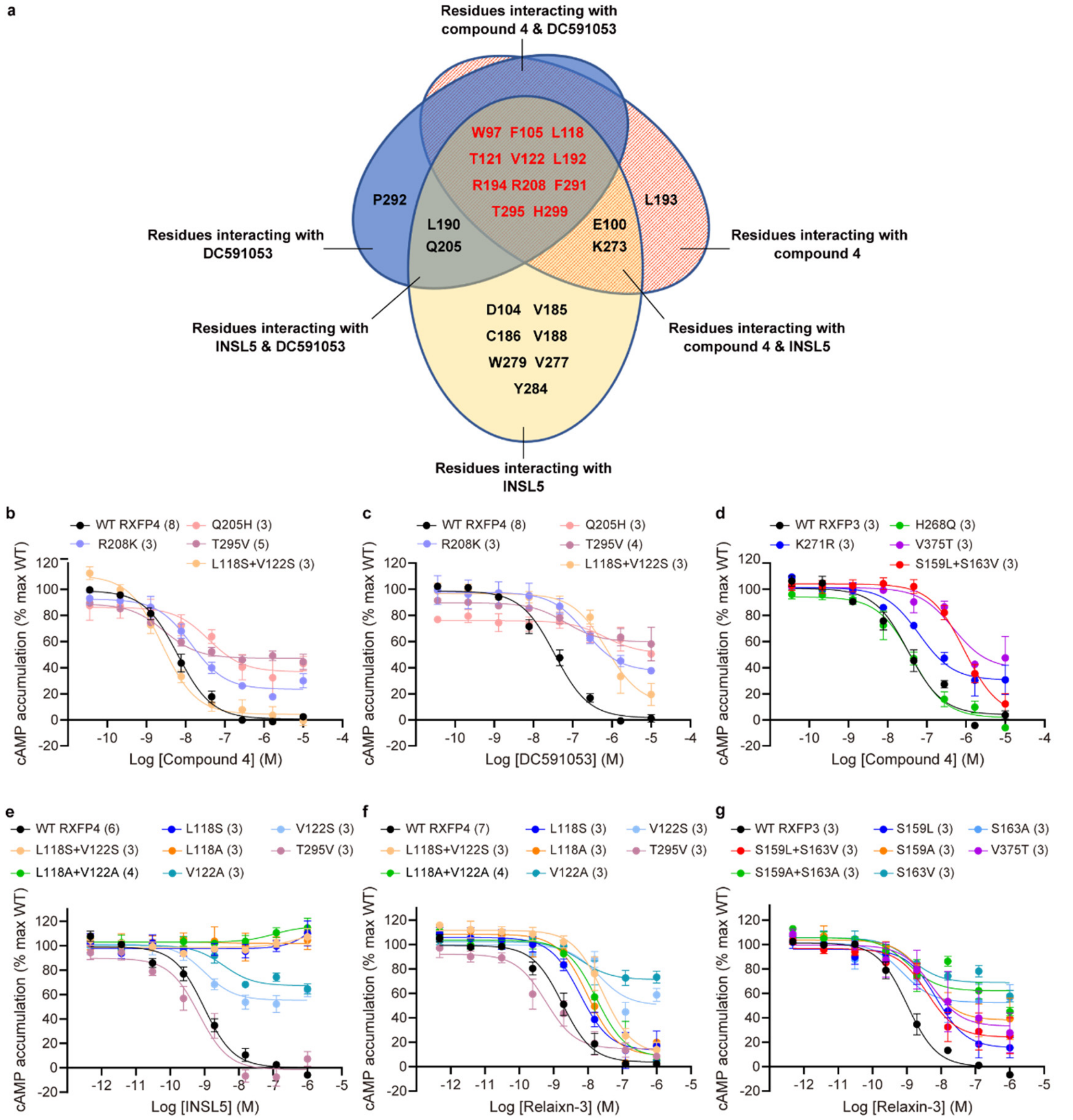

Supplementary Figure 10. Peptidomimetic agonism and key residues on receptor subtype selectivity. a, RXFP4 residues are categorized according to their interactions with the three ligands. **b-d,** Effects of amino acid switch in equivalent positions between RXFP4 and RXFP3 around the ligand-binding pocket on compound 4 **(b)** and DC591053 **(c)** induced cAMP accumulation in RXFP4 as well as on compound 4 induced cAMP accumulation in RXFP3 **(d)**. **e-f,** Effects of INSL5 **(e)** and relaxin-3 **(f)** on cAMP accumulation in WT and mutant RXFP4s. **g,** Effects of relaxin-3 on cAMP accumulation in WT and mutant RXFP3. INSL5 was totally inactive in RXFP4 single mutants L118$^{3.29}$S and L118$^{3.29}$A as well as double mutants L118$^{3.29}$S+V122$^{3.33}$S and L118$^{3.29}$A+V122$^{3.33}$A, where relaxin-3 retained partial activity although the curves shifted to the right (by 3.2-fold, 5.4-fold, 21.8-fold and 9.7-fold, respectively). For comparison, relaxin-3 activated S159$^{3.29}$A, S159$^{3.29}$L, S159$^{3.29}$L+S163$^{3.33}$V and S159$^{3.29}$A+S163$^{3.33}$A in RXFP3 albeit with reduced potencies. T295$^{7.39}$V did not destroy the response of RXFP4 to INSL5 and relaxin-3, but

V375$^{7.39}$T in RXFP3 impaired both the potency (by 5.1-fold) and E_{max} (66.5% of the WT) of relaxin-3 (INSL5 was inactive in WT and all the five RXFP3 mutants). Therefore, S159$^{3.29}$, S163$^{3.33}$ and V375$^{7.39}$ in RXFP3 and L118$^{3.29}$, V122$^{3.33}$ and T295$^{7.39}$ in RXFP4 are likely involved in RXFP3 *vs.* RXFP4 subtype selectivity, consistent with the observations in RXFP3/RXFP4 chimeric receptor studies[4]. Data were shown as means ± S.E.M. of at least three independent experiments. The numbers of independent experiments are shown in the parentheses. Supplementary Tables 4 and 5 provide detailed statistical evaluation such as *P* values and numbers of independent experiments (*n*). Source data are provided as a Source Data file.

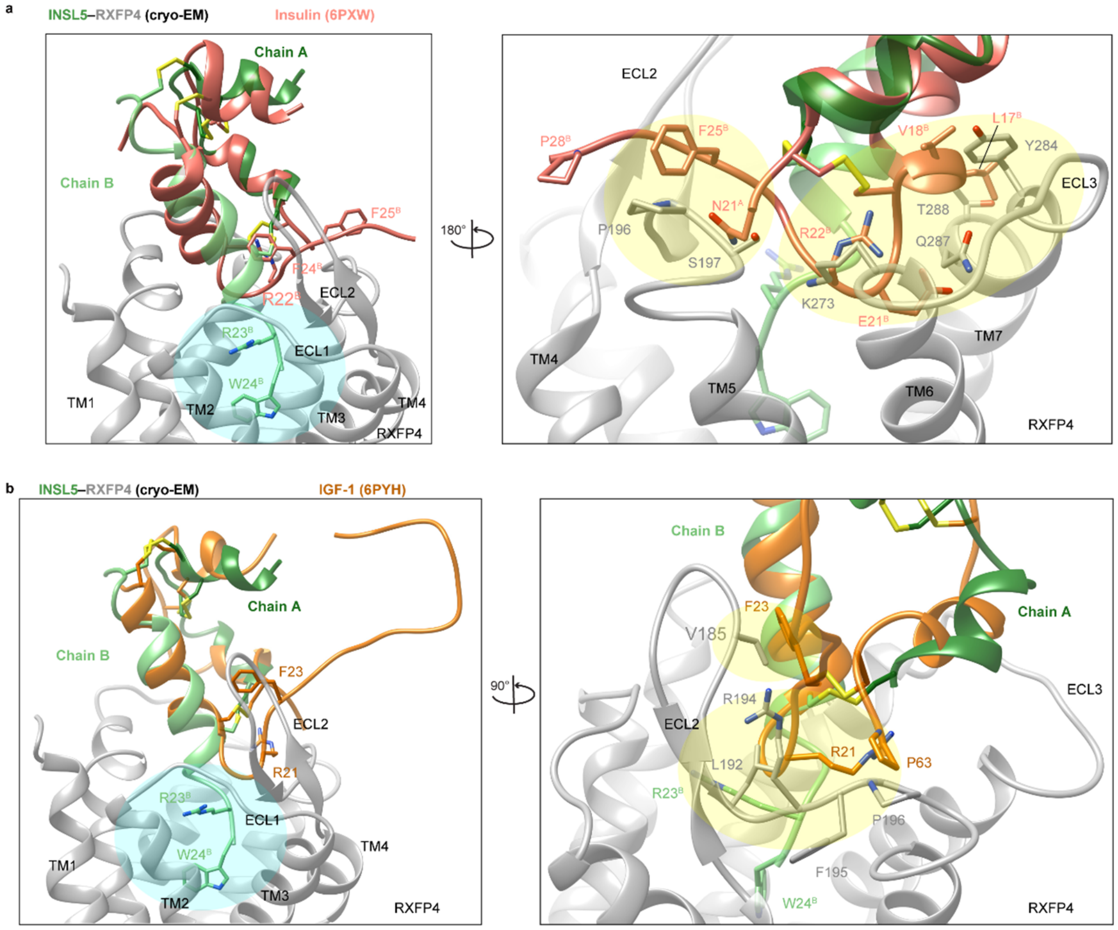

Supplementary Figure 11. Superimposition of INSL5 from the INSL5–RXFP4–G_i complex structure with insulin or IGF-1. a, Insulin (salmon, PDB code: 6PXW) was superimposed on the INSL5 (green) from the cryo-EM structure of INSL5–RXFP4–G_i using the Cα atoms. The three disulfide bonds and key residues in the peptide-receptor interface are shown in sticks. Compared to the endogenous agonist INSL5, the aligned insulin loses multiple potent interactions (cyan-shaded region) and causes significant steric clashes (yellow-shaded region) with RXFP4 (gray). **b,** IGF-1 (orange, PDB code: 6PYH) was superimposed on the INSL5 (green) from the cryo-EM structure of INSL5–RXFP4–G_i using the Cα atoms. Compared to the endogenous agonist INSL5, the aligned IGF-1 loses multiple potent interactions (cyan-shaded region) and causes significant steric clashes (yellow-shaded region) with RXFP4 (gray).

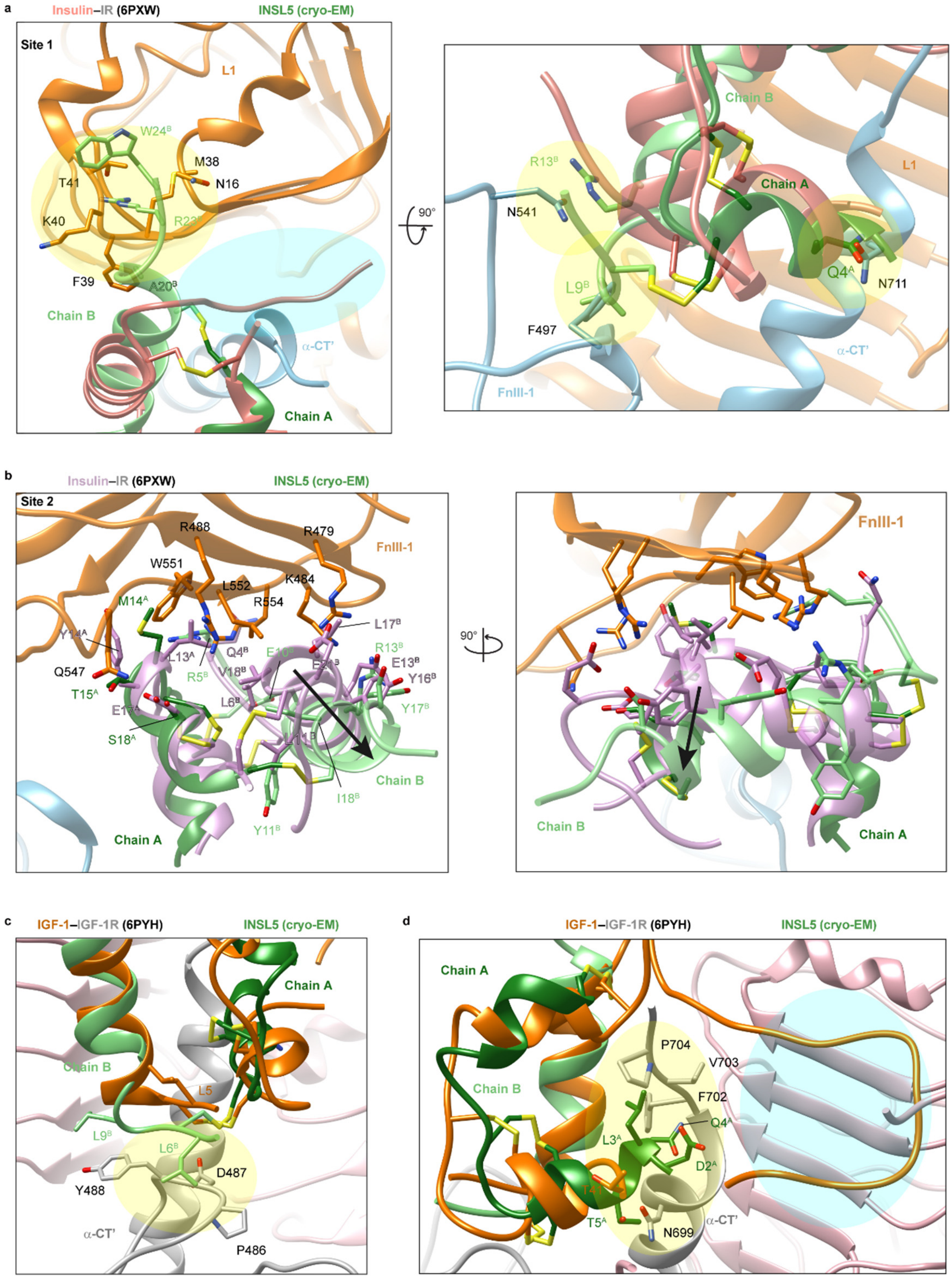

Supplementary Figure 12. Superimposition of INSL5 to insulin or IGF-1 in complex with cognate receptors. a-b, INSL5 (green) was superimposed on the insulin at site 1 (salmon, **a**) or at site 2 (plum, **b**) from the cryo-EM structure of insulin–insulin receptor (PDB code: 6PXW) using the Cα atoms. The three disulfide bonds and key residues in the peptide-receptor interface are shown in sticks. Compared to the endogenous agonist insulin, the aligned INSL5 loses multiple potent interactions (cyan-shaded region) and causes significant steric clashes (yellow-shaded region) with

insulin receptor. **c,** INSL5 (green) was superimposed on the IGF-1 (orange) from the cryo-EM structure of IGF-1–IGF-1R (PDB code: 6PYH) using the Cα atoms. The three disulfide bonds and key residues in the peptide-receptor interface are shown in sticks. **d,** Compared to the endogenous agonist IGF-1, the aligned INSL5 loses multiple potent interactions (cyan-shaded region) and causes significant steric clashes (yellow-shaded region) with IGF-1R.

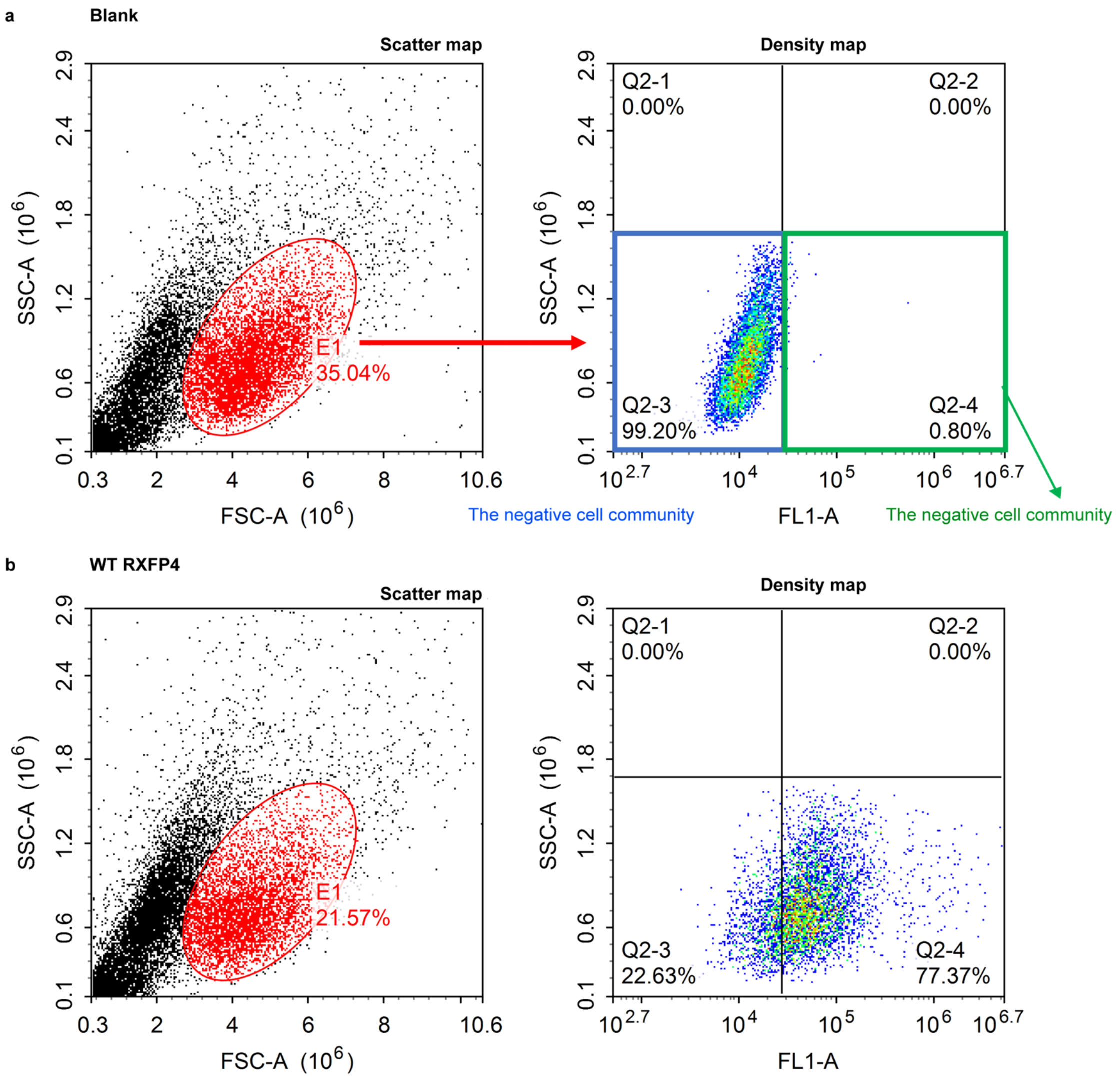

Supplementary Figure 13. Gating strategy of the cell surface expression assay. Circle a gate E1 in the scatter map (red circle) and the cells shown in the density map are all the cells in the gate E1 of the scatter map. Fluorescence signal intensity (FITC) is presented by density map. With the blank sample (no receptor transfection) as the reference value of background fluorescence signal (**a**), the "quadrant gate" divides the fluorescence signal density map into four quadrants. The third quadrant represents the negative cell community, while the fourth quadrant represents the positive cell community. The expression value of wild-type (WT) receptor (**b**) can be calculated as follows: (M(Q2-4)-M(Q2-3)) × (Q2-4% Parent). The calculation of receptor mutants is the same as that of the WT receptor, which was then normalized with the WT receptor to calculate the relative expression value of the mutants.

Supplementary Table 1 Cryo-EM data collection, refinement and validation statistics.

	**INSL5–RXFP4 (1-374)–G$_i$**	**Compound 4–RXFP4 (1-374)–G$_i$**	**DC591053–RXFP4 (1-374)–G$_i$**
**Data collection and processing**			
Magnification	46,685	46,685	46,685
Voltage (kV)	300	300	300
Electron exposure ($e^{-}/Å^2$)	80	80	80
Defocus range (μm)	-1.2 to -2.2	-1.2 to -2.2	-1.2 to -2.2
Pixel size (Å)	1.071	1.071	1.071
Symmetry imposed	Cl	Cl	Cl
Initial particle images (no.)	10,618,534	4,796,219	8,996,005
Final particle images (no.)	524,035	243,800	225,327
Map resolution (Å)	3.19	3.03	2.75
FSC threshold	0.143	0.143	0.143
Map resolution range (Å)	2.5-5.0	2.5-5.0	2.5-5.0
**Refinement**			
Initial model used (PDB code)	PDB codes 7F2O and 6D9H	PDB codes 7F2O and 6D9H	PDB codes 7F2O and 6D9H
Model resolution (Å)	3.3	3.2	3.1
FSC threshold	0.5	0.5	0.5
Model resolution range (Å)	2.5-5.0	2.5-5.0	2.5-5.0
Map sharpening B factor ($Å^2$)	-163.30	-96.90	-106.50
Model composition			
Non-hydrogen atoms	8980	8731	8761
Protein residues	1154	1118	1122
B factors ($Å^2$)			
Protein	93.61	80.83	78.02
Ligand	—	70.68	77.67
R.m.s. deviations			
Bond lengths (Å)	0.004	0.004	0.004
Bond angles (°)	0.599	0.552	0.666
Validation			
MolProbity score	1.84	1.69	1.79

Clash score	9.24	8.75	8.03
Poor rotamers (%)	0.00	0.00	0.00
Ramachandran plot			
Favored (%)	94.96	96.61	94.98
Allowed (%)	5.04	3.39	5.02
Disallowed (%)	0.00	0.00	0.00
Rama-Z score	0.71	0.16	0.78

Supplementary Table 2 Interactions of INSL5, compound 4 and DC591053 with RXFP4.

RXFP4	INSL5	Compound 4	DC591053
W97$^{2.60}$	Stacking	Stacking	Stacking
E100$^{2.63}$	Salt bridge	Salt bridge	——
D104$^{2.67}$	Hydrogen bond	——	——
F105^{ECL1}	Stacking	Stacking	Stacking
L118$^{3.29}$	Hydrophobic contact	Hydrophobic contact	Hydrophobic contact
T121$^{3.32}$	Hydrogen bond	Hydrophobic contact	Hydrophobic contact
V122$^{3.33}$	Hydrophobic contact	Hydrophobic contact	Hydrophobic contact
V185^{ECL2}	Hydrophobic contact	——	——
C186^{ECL}	Hydrophobic contact	——	——
V188^{ECL2}	Hydrophobic contact	——	——
L190^{ECL2}	Hydrophobic contact	——	Hydrophobic contact
L192$^{45.51}$	Hydrophobic contact	Hydrophobic contact	Hydrophobic contact
L193$^{45.52}$	——	Hydrophobic contact<br>Hydrogen bond	——
R194^{ECL2}	Weak hydrogen bond	Hydrogen bond	Stacking
Q205$^{5.39}$	Hydrogen bond	——	Hydrogen bond
R208$^{5.42}$	Salt bridge<br>Stacking	Stacking	Hydrogen bond<br>Stacking
K273$^{6.62}$	Salt bridge<br>Hydrophobic contact	Hydrogen bond	——
V277^{ECL3}	Hydrophobic contact	——	——
W279^{ECL3}	Hydrophobic contact	——	——
Y284$^{7.28}$	Hydrophobic contact	——	——
F291$^{7.35}$	Stacking	Stacking	Stacking
P292$^{7.36}$	——	——	Hydrophobic contact
T295$^{7.39}$	Hydrophobic contact	Hydrogen bond	Hydrogen bond
H299$^{7.43}$	Stacking	Hydrogen bond	Stacking

Supplementary Table 3. Ligand-mediated inhibition of forskolin-induced cAMP accumulation.

Receptor	INSL5					Compound 4					DC591053				
	pEC_{50}		E_{max} (% WT)		*n*	*p*EC_{50}		E_{max} (% WT)		*n*	*p*EC_{50}		E_{max} (% WT)		*n*
	Means ± S.E.M.	*P* value	Means ± S.E.M.	*P* value		Means ± S.E.M.	*P* value	Means ± S.E.M.	*P* value		Means ± S.E.M.	*P* value	Means ± S.E.M.	*P* value	
WT RXFP4	9.07 ± 0.07	—	99.76 ± 2.15	—	3	7.94 ± 0.08	—	99.99 ± 2.85	—	3	7.68 ± 0.09	—	99.14 ± 2.97	—	3
HA-H10-Bril-RXFP4 -15AA-LgBiT	8.94 ± 0.08	0.8616	110.76 ± 2.64	0.7961	3	7.74 ± 0.07	0.8060	110.40 ± 2.36	0.8141	3	7.14 ± 0.08	0.8601	108.57 ± 2.51	0.8329	3

All data were fitted with a three-parameter logistic curve to obtain *p*EC_{50} and E_{max} values. The assay was performed in transiently transfected HEK293T cells. Data are presented as means ± S.E.M. of three independent experiments (n = 3) performed in quadruplicate. Statistical analysis was performed using a two-tailed Student's *t*-test and no significance was found among the values. The *P* value was defined as: $^*P < 0.05$, $^{**}P < 0.01$, $^{***}P < 0.001$ and $^{****}P < 0.0001$. WT, wild-type.

Supplementary Table 4. Cell surface expression and effects of residue mutation on ligand-mediated inhibition of forskolin-induced cAMP accumulation.

Cell surface expression of receptor mutations				
**Receptor**	**Mutation**	**Cell surface expression (% WT)**	***P* value**	***n***
RXFP4	WT	100	——	7
	W97A	59.76 ± 0.21****	<0.0001	3
	E100A	81.83 ± 4.56	0.1014	4
	D104A	38.82 ± 1.34****	<0.0001	3
	F105A	69.26 ± 2.69**	0.0016	3
	T121A	40.11 ± 2.25****	<0.0001	3
	R194A	29.81 ± 1.01****	<0.0001	3
	Q205A	80.26 ± 2.04	0.0569	4
	R208A	27.07 ± 1.09****	<0.0001	3
	K273A	35.92 ± 2.69****	<0.0001	3
	W279A	151.25 ± 6.66****	<0.0001	5
	Y284A	123.5 ± 8.30**	0.0059	5
	H299A	84.87 ± 8.49	0.1966	5
	L118S+V122S	24.79 ± 2.68****	<0.0001	3
	Q205H	91.09 ± 1.43	0.8954	4
	R208K	68.61 ± 4.73****	<0.0001	5
	T295V	66.15 ± 7.26****	<0.0001	5
RXFP3	WT	100	——	3
	S159L+S163V	78.75 ± 2.54***	0.0003	3
	H268Q	96.03 ± 2.79	0.5787	3
	K271R	97.86 ± 1.15	0.9076	3
	V375T	95.63 ± 3.30	0.5022	3

Effects of residue mutation on INSL5-mediated inhibition of forskolin-induced cAMP accumulation						
**Receptor**	**Mutation**	***p*EC$_{50}$**		**E$_{max}$ (% WT)**		***n***
		**Mean ± S.E.M.**	***P* value**	**Mean ± S.E.M.**	***P* value**	
RXFP4	WT	9.12 ± 0.06	——	99.88 ± 2.28	——	6
	W97A	7.81 ± 0.18***	0.0008	61.33 ± 5.34****	<0.0001	3
	E100A	N.A.	——	N.A.	——	3
	D104A	8.79 ± 0.25	0.8176	59.09 ± 6.41****	<0.0001	3
	F105A	8.00 ± 0.11**	0.0042	80.16 ± 4.08	0.0571	3
	T121A	N.A.	——	N.A.	——	4
	R194A	8.78 ± 0.17	0.7863	79.76 ± 6.06	0.0502	3
	Q205A	8.40 ± 0.30	0.1002	50.94 ± 6.54****	<0.0001	3
	R208A	N.A.	——	N.A.	——	3
	K273A	8.45 ± 0.29	0.0940	58.10 ± 7.38****	<0.0001	4

	W279A	8.73 ± 0.17	0.6679	43.30 ± 3.29****	<0.0001	3
	Y284A	8.74 ± 0.19	0.6174	49.25 ± 4.06****	<0.0001	4
	H299A	9.02 ± 0.29	0.9994	30.77 ± 3.77****	<0.0001	3

Effects of residue mutation on compound 4-mediated inhibition of forskolin-induced cAMP accumulation

Receptor	Mutation	*p*EC50		Emax (% WT)		*n*
		Mean ± S.E.M.	*P* value	Mean ± S.E.M.	*P* value	
RXFP4	WT	8.20 ± 0.07	——	98.22 ± 3.01	——	8
	W97A	N.A.	——	N.A.	——	—
	E100A	N.A.	——	N.A.	——	—
	F105A	7.81 ± 0.21	0.5144	60.11 ± 5.70****	<0.0001	3
	T121A	6.90 ± 0.30****	<0.0001	59.77 ± 8.29****	<0.0001	3
	R194A	7.33 ± 0.24 **	0.0056	78.96 ± 8.75	0.0619	3
	R208A	7.38 ± 0.17**	0.0094	63.76 ± 4.94***	0.0002	3
	K273A	7.33 ± 0.19**	0.0056	76.06 ± 6.54*	0.0227	3
	H299A	N.A.	——	N.A.	——	3
	L118S+V122S	8.60 ± 0.10	0.5000	106.23 ± 4.75	0.8663	3
	Q205H	7.41 ± 0.32*	0.0135	49.35 ± 7.02****	<0.0001	3
	R208K	7.85 ± 0.13	0.6570	69.09 ± 4.02**	0.0016	3
	T295V	8.55 ± 0.15	0.4573	41.58 ± 2.93****	<0.0001	5
RXFP3	WT	7.48 ± 0.12	——	96.70 ± 5.12	——	3
	S159L+S163V	6.05 ± 0.12***	0.0006	101.29 ± 6.72	0.4990	3
	H268Q	7.39 ± 0.11	0.9792	92.52 ± 4.71	0.2208	3
	K271R	7.21 ± 0.17	0.8018	70.86 ± 5.48****	<0.0001	3
	V375T	6.33 ± 0.24**	0.0031	62.11 ±7.78****	<0.0001	3

Effects of residue mutation on DC591053-mediated inhibition of forskolin-induced cAMP accumulation

Receptor	Mutation	*p*EC50		Emax (% WT)		*n*
		Mean ± S.E.M.	*P* value	Mean ± S.E.M.	*P* value	
RXFP4	WT	7.46 ± 0.07	——	97.18 ± 3.11	——	8
	W97A	7.25 ± 0.10	0.9961	108.65 ± 10.04	0.6745	3
	E100A	6.97 ± 0.18	0.6133	79.27 ± 6.72	0.1533	3
	F105A	6.77 ± 0.15	0.1851	57.39 ± 4.17****	<0.0001	3
	T121A	6.14 ± 0.41***	0.0007	31.53 ± 7.09****	<0.0001	3
	R194A	6.55 ± 0.19*	0.0339	81.10 ± 7.60	0.2549	3
	R208A	6.58 ± 0.16*	0.0431	52.09 ± 4.26****	<0.0001	3
	K273A	5.73 ± 0.18****	<0.0001	68.51 ± 8.34**	0.0035	3
	H299A	8.44 ± 0.24*	0.0164	36.04 ± 4.01****	<0.0001	3
	L118S+V122S	6.14 ± 0.18***	0.0007	84.85 ± 8.36	0.5869	3
	Q205H	6.17 ± 0.46***	0.001	25.10 ± 6.36****	<0.0001	3
	R208K	6.69 ± 0.22	0.1016	60.43 ± 6.52***	0.0001	3
	T295V	7.19 ± 0.33	0.9657	30.00 ± 4.54****	<0.0001	4

Inhibition of forskolin-induced cAMP accumulation was performed in HEK293T cells transiently transfected with WT and mutant receptors. All the mutant constructs were modified by single-point mutation in the setting of the WT receptor. cAMP accumulation data were analyzed using a three-parameter logistic equation to determine $p\mathrm{EC}_{50}$ and E_{max} values. E_{max} values for mutants are defined as the window between the maximal response and vehicle control (no ligand) and expressed as a percentage of the WT. Cell surface expression was assessed by flow cytometry to detect the N-terminal Flag epitope label on the receptors and normalized to the WT receptor (shown as percentage). Data shown are means ± S.E.M. of at least three independent experiments. One-way ANOVA were used to determine statistical difference (*$P< 0.05$, **$P< 0.01$, ***$P< 0.001$ and ****$P< 0.0001$). *n*, sample size; the number of independent experiments. N.A., not active.

Supplementary Table 5. Effects of key residues mutation on receptor subtype selectivity.

Receptor	Mutation	INSL5					Relaxin-3				
		*p*EC50		Emax (% WT)		*n*	*p*EC50		Emax (% WT)		*n*
		Means ± S.E.M.	*P* value	Means ± S.E.M.	*P* value		Means ± S.E.M.	*P* value	Means ± S.E.M.	*P* value	
RXFP4	WT	9.02 ± 0.09	——	98.86 ± 3.65	——	6	8.74 ± 0.10	——	95.70 ± 4.30	——	7
	T295V	9.16 ± 0.17	0.8687	91.17 ± 6.47	0.5243	3	9.23 ± 0.20	0.2688	77.70 ± 6.32	0.2278	3
	L118S+V122S	N.A.	——	N.A.	——	3	7.40 ± 0.14****	<0.0001	106.69 ± 7.69	0.7224	3
	L118A+V122A	N.A.	——	N.A.	——	4	7.75 ± 0.18**	0.0011	96.13 ± 8.52	>0.9999	4
	L118A	N.A.	——	N.A.	——	3	8.01 ± 0.14*	0.0361	99.73 ± 6.19	0.9975	3
	L118S	N.A.	——	N.A.	——	3	8.23 ± 0.17	0.2305	92.78 ± 6.88	0.9996	3
	V122A	8.44 ± 0.23	0.0728	33.20 ± 3.27****	<0.0001	3	8.20 ± 0.31	0.1719	31.24 ± 4.24****	<0.0001	3
	V122S	8.99 ± 0.26	0.9995	45.95 ± 4.97****	<0.0001	3	7.69 ± 0.27**	0.0016	49.66 ± 6.44***	0.0001	3
RXFP3	WT	N.D.	——	N.D.	——	——	8.99 ± 0.09	——	101.40 ± 3.62	——	3
	V375T	N.D.	——	N.D.	——	——	8.28 ± 0.41	0.3706	66.57 ± 11.87*	0.0169	3
	S159L+S163V	N.D.	——	N.D.	——	——	8.41 ± 0.23	0.5638	72.69 ± 7.22	0.0562	3
	S159A+S163A	N.D.	——	N.D.	——	——	8.96 ± 0.38	0.9999	43.57 ± 6.93***	0.0002	3
	S159L	N.D	——	N.D.	——	——	8.13 ± 0.17	0.2088	81.00 ± 6.00	0.2470	3
	S159A	N.D.	——	N.D.	——	——	8.41 ± 0.25	0.5624	65.67 ± 7.00*	0.0141	3
	S163V	N.D.	——	N.D.	——	——	8.87 ± 0.26	0.9996	36.91 ± 4.14****	<0.0001	3
	S163A	N.D.	——	N.D.	——	——	9.12 ± 0.34	0.9995	48.85 ± 6.78***	0.0005	3

Inhibition of forskolin-induced cAMP accumulation was performed in HEK293T cells transiently transfected with WT and mutant receptors. All the mutant constructs were modified by single-point mutation in the setting of the WT receptor. cAMP accumulation data were analyzed using a three-parameter logistic equation to determine pEC_{50} and E_{max} values. E_{max} values for mutants are defined as the window between the maximal response and vehicle control (no ligand) and expressed as a percentage of the WT. Data shown are means ± S.E.M. of at least three independent experiments. One-way ANOVA were used to determine statistical difference (*P< 0.05, **P< 0.01, ***P< 0.001 and ****P< 0.0001). *n*, sample size; the number of independent experiments. N.A., not active; N.D., not determined.

Supplementary Table 6 | Primers used in this study, related to Figures 2 and 3, Supplementary Figures 3 and 10 and Supplementary Tables 3, 4 and 5.

**Oligonucleotide name**	**Oligonucleotide sequence (5'-3')**	**Cloning method**	**Product**
Insert-fragment-forward	AGATCTGCGCCGCGATCGCCCAAAATGAAGACGATCATCGCC	Homologous recombination	pCMV6-HA-H10-BRIL-RXFP4(1-374)-LgBiT
Insert-fragment-reverse	CTATGACCGCGGCCGGCCGTTTAGCTGTTGATGGTTACTCGGAA		
Linear-pCMV6-forward	ACGGCCGGCCGCGGTCAT		
Linear-pCMV6-reverse	GGCGATCGCGGCGCAGAT		
Add-RXFP4-Flag-forward	CGATCGCCATGGACTACAAAGACGATGACGACAAGCCCACACTCAATACT	Site-directed mutagenesis	pCMV6-Flag-RXFP4
Add-RXFP4-Flag-reverse	GAGTGTGGGCTTGTCGTCATCGTCTTTGTAGTCCATGGCGATCGCGGCGC		
Add-RXFP3-Flag-forward	CTTGCCATGGACTACAAAGACGATGACGACAAGCAGATGGCCGATGCAGCCAC		pCMV6-Flag-RXFP3
Add-RXFP3-Flag-reverse	CTTGTCGTCATCGTCTTTGTAGTCCATGGCAAGCTTGGCGGCAGATCTC		
W97A-forward	GCACTCACTCTCCCCTTTGCGGCAGCCGAG	Site-directed mutagenesis	pCMV6-RXFP4(1-374)-W97A
W97A-reverse	CTCGGCTGCCGCAAAGGGGAGAGTGAGTGC		
E100A-forward	TTTTGGGCAGCCGCGTCGGCACTGGAC		pCMV6-RXFP4(1-374)-E100A
E100A-reverse	GTCCAGTGCCGACGCGGCTGCCCAAAA		
D104A-forward	GAGTCGGCACTGGCCTTTCACTGGCCC		pCMV6-RXFP4(1-374)-D104A
D104A-reverse	GGGCCAGTGAAAGGCCAGTGCCGACTC		
F105A-forward	GAGTCGGCACTGGACGCTCACTGGCCCTTCGG		pCMV6-RXFP4(1-374)-F105A
F105A-reverse	CCGAAGGGCCAGTGAGCGTCCAGTGCCGACTC		
T121A-forward	TGGTTCTGACGGCCGCTGTCCTCAACGT		pCMV6-RXFP4(1-374)-T121A
T121A-reverse	GACGTTGAGGACAGCGGCCGTCAGAACCA		
R194A-forward	GCCTTTGCCTGCTGGCTTTCCCCAGCAGGT		pCMV6-RXFP4(1-374)-R194A
R194A-reverse	ACCTGCTGGGGAAAGCCAGCAGGCAAAGGC		
Q205A-forward	GCTGGGGGCCTACGCGCTGCAGAGGGTG		pCMV6-RXFP4(1-374)-Q205A
Q205A-reverse	CACCCTCTGCAGCGCGTAGGCCCCCAGC		
R208A-forward	CCTACCAGCTGCAGGCGGTGGTGCTGGCTT		pCMV6-RXFP4(1-374)-R208A
R208A-reverse	AAGCCAGCACCACCGCCTGCAGCTGGTAGG		

K273A-forward	TGGGGTGTCCTGGTGGCGTTTGACCTGGTGCC		pCMV6-RXFP4(1-374)-K273A
K273A-reverse	GGCACCAGGTCAAACGCCACCAGGACACCCCA		
W279A-forward	GAAGTTTGACCTGGTGCCCGCGAACAGTACTTTCTATACTA		pCMV6-RXFP4(1-374)-W279A
W279A-reverse	TAGTATAGAAAGTACTGTTCGCGGGCACCAGGTCAAACTTC		
Y284A-forward	GCCCTGGAACAGTACTTTCGCTACTATCCAGACGTATGTC		pCMV6-RXFP4(1-374)-Y284A
Y284A-reverse	GACATACGTCTGGATAGTAGCGAAAGTACTGTTCCAGGGC		
T295A-forward	TGTCTTCCCTGTCACTGCTTGCTTGGCACACAG		pCMV6-RXFP4(1-374)-T295A
T295A-reverse	CTGTGTGCCAAGCAAGCAGTGACAGGGAAGACA		
H299A-forward	GTCACTACTTGCTTGGCAGCCAGCAATAGCTGCCTCAA		pCMV6-RXFP4(1-374)-H299A
H299A-reverse	TTGAGGCAGCTATTGCTGGCTGCCAAGCAAGTAGTGAC		
Q205H-forward	GGGGGCCTACCATCTGCAGAGGGTG		pCMV6-RXFP4(1-374)-Q205H
Q205H-reverse	CACCCTCTGCAGATGGTAGGCCCCC		
R208K-forward	GGCCTACCAGCTGCAGAAGGTGGTGCT		pCMV6-RXFP4(1-374)-R208K
R208K-reverse	AGCACCACCTTCTGCAGCTGGTAGGCC		
T295V-forward	GCCCTCTGCAAGATGGTTTCGACGGCCACTAGCCTCAACGTCTATGC		pCMV6-RXFP4(1-374)-T295V
T295V-reverse	GCATAGACGTTGAGGCTAGTGGCCGTCGAAACCATCTTGCAGAGGGC		
L118S+V122S-forward	GCCCTCTGCAAGATGGTTTCGACGGCCACTAGCCTCAACGTCTATGC		pCMV6-RXFP4(1-374)-L118S+V122S
L118S+V122S-reverse	GCATAGACGTTGAGGCTAGTGGCCGTCGAAACCATCTTGCAGAGGGC		
L118A+V122A-forward	GTGCCCTCTGCAAGATGGTTGCGACGGCCACTG		pCMV6-RXFP4(1-374)-L118A+V122A
L118A+V122A-reverse	CAGTGGCCGTCGCAACCATCTTGCAGAGGGCAC		
L118A-forward	TTCTGACGGCCACTGCCCTCAACGTCTATGCCAGCA		pCMV6-RXFP4(1-374)-L118A
L118A-reverse	ATAGACGTTGAGGGCAGTGGCCGTCAGAACCATCTT		
V122A-forward	GTGCCCTCTGCAAGATGGTTTCGACGGCCACTG		pCMV6-RXFP4(1-374)-L118S
V122A-reverse	CAGTGGCCGTCGAAACCATCTTGCAGAGGGCAC		

L118S-forward	TCTGACGGCCACTAGCCTCAACGTCTATGCCAGCATC		pCMV6-RXFP4(1-374)-V122A
L118S-reverse	GGCATAGACGTTGAGGCTAGTGGCCGTCAGAACCATCT		pCMV6-RXFP4(1-374)-V122A
V122S-forward	AAGATGGTTGCGACGGCCACTGCCCTCAACGTC		pCMV6-RXFP4(1-374)-V122S
V122S-reverse	TTGAGGGCAGTGGCCGTCGCAACCATCTTGCA		pCMV6-RXFP4(1-374)-V122S
H268Q-forward	TGGCTGGGCCTCTACCAGTCGCAGAAG	Site-directed mutagenesis	pCMV6-RXFP3(1-469)-H268Q
H268Q-reverse	CTTCTGCGACTGGTAGAGGCCCAGCCA	Site-directed mutagenesis	pCMV6-RXFP3(1-469)-H268Q
K271R-forward	GGCCTCTACCACTCGCAGAGGGTGCTGCTG	Site-directed mutagenesis	pCMV6-RXFP3(1-469)-K271R
K271R-reverse	CAGCAGCACCCTCTGCGAGTGGTAGAGGCC	Site-directed mutagenesis	pCMV6-RXFP3(1-469)-K271R
V375T-forward	GCGTTCCCTGTGAGCACGTGCCTAGCGCACTC	Site-directed mutagenesis	pCMV6-RXFP3(1-469)-V375T
V375T-reverse	GAGTGCGCTAGGCACGTGCTCACAGGGAACGC	Site-directed mutagenesis	pCMV6-RXFP3(1-469)-V375T
S159L+S163V-forward	GCCATGTGTAAGATCGTGTTAATGGTGACGGTCATGAACATGTACGCCAGC	Site-directed mutagenesis	pCMV6-RXFP3(1-469)-S159L + S163L
S159L+S163V-reverse	GCTGGCGTACATGTTCATGACCGTCACCATTAACACGATCTTACACATGGC	Site-directed mutagenesis	pCMV6-RXFP3(1-469)-S159L + S163L
S159L+S163A-forward	TAAGATCGTGGCCATGGTGACGGCCATGAACATGTACGCC	Site-directed mutagenesis	pCMV6-RXFP3(1-469)-S159A + S163A
S159A+S163A-reverse	ATGTTCATGGCCGTCACCATGGCCACGATCTTACACATGG	Site-directed mutagenesis	pCMV6-RXFP3(1-469)-S159A + S163A
S159A-forward	CCATGTGTAAGATCGTGGCCATGGTGACGTCCATG	Site-directed mutagenesis	pCMV6-RXFP3(1-469)-S159A
S159A-reverse	CATGGACGTCACCATGGCCACGATCTTACACATGG	Site-directed mutagenesis	pCMV6-RXFP3(1-469)-S159A
S163A-forward	TCCATGGTGACGGCCATGAACATGTACGCCAGCGTGT	Site-directed mutagenesis	pCMV6-RXFP3(1-469)-S163A
S163A-reverse	GCGTACATGTTCATGGCCGTCACCATGGACACGATCTT	Site-directed mutagenesis	pCMV6-RXFP3(1-469)-S163A
S159L-forward	GCCATGTGTAAGATCGTGTTAATGGTGACGTCCATGAACA	Site-directed mutagenesis	pCMV6-RXFP3(1-469)-S159L
S159L-reverse	TGTTCATGGACGTCACCATTAACACGATCTTACACATGGC	Site-directed mutagenesis	pCMV6-RXFP3(1-469)-S159L
S163V-forward	ATCGTGTCCATGGTGACGGTCATGAACATGTACGCCAG	Site-directed mutagenesis	pCMV6-RXFP3(1-469)-S163V
S163V-reverse	CTGGCGTACATGTTCATGACCGTCACCATGGACACGAT	Site-directed mutagenesis	pCMV6-RXFP3(1-469)-S163V

Supplementary Table 7. Details of restraints applied during MD simulations.

**Stage**	**Time step**	**Simulation time**	**Restrain**
Heating	1 fs	1 ns	Position harmonic restrain (40 kJ·mol^{-1}·Å^{-2}) for the backbone non-hydrogen atoms of protein and peptide;<br>Position restrain (20 kJ·mol^{-1}·Å^{-2}) for the sidechain non-hydrogen atoms of protein and peptide;<br>Planar harmonic restraint (10 kJ·mol^{-1}·Å^{-2}) for the phosphorus atom of POPC along the Z-axis;<br>Dihedral restraint (1000 kJ·mol^{-1}·rad^{-2}) for two dihedrals (C28-C29-C210-C211 and C1-C3-C2-O21).
Step6.1	1 fs	5 ns	Position harmonic restrain (40 kJ·mol^{-1}·Å^{-2}) for the backbone non-hydrogen atoms of protein and peptide;<br>Position restrain (20 kJ·mol^{-1}·Å^{-2}) for the sidechain non-hydrogen atoms of protein and peptide;<br>Planar harmonic restraint (10 kJ·mol^{-1}·Å^{-2}) for the phosphorus atom of POPC along the Z-axis;<br>Dihedral restraint (1000 kJ·mol^{-1}·rad^{-2}) for two dihedrals (C28-C29-C210-C211 and C1-C3-C2-O21).
Step6.2	1 fs	5 ns	Position harmonic restrain (20 kJ·mol^{-1}·Å^{-2}) for the backbone non-hydrogen atoms of protein and peptide;<br>Position restrain (10 kJ·mol^{-1}·Å^{-2}) for the sidechain non-hydrogen atoms of protein and peptide;<br>Planar harmonic restraint (4 kJ·mol^{-1}·Å^{-2}) for the phosphorus atom of POPC along the Z-axis;<br>Dihedral restraint (400 kJ·mol^{-1}·rad^{-2}) for two dihedrals (C28-C29-C210-C211 and C1-C3-C2-O21).
Step6.3	2 fs	10 ns	Position harmonic restrain (10 kJ·mol^{-1}·Å^{-2}) for the backbone non-hydrogen atoms of protein and peptide;<br>Position restrain (5 kJ·mol^{-1}·Å^{-2}) for the sidechain non-hydrogen atoms of protein and peptide;<br>Planar harmonic restraint (4 kJ·mol^{-1}·Å^{-2}) for the phosphorus atom of POPC along the Z-axis;

			Dihedral restraint (200 kJ·mol^{-1}·rad^{-2}) for two dihedrals (C28-C29-C210-C211 and C1-C3-C2-O21).
Step6.4	2 fs	10 ns	Position harmonic restrain (5 kJ·mol^{-1}·Å^{-2}) for the backbone non-hydrogen atoms of protein and peptide;<br>Position restrain (2 kJ·mol^{-1}·Å^{-2}) for the sidechain non-hydrogen atoms of protein and peptide;<br>Planar harmonic restraint (2 kJ·mol^{-1}·Å^{-2}) for the phosphorus atom of POPC along the Z-axis;<br>Dihedral restraint (200 kJ·mol^{-1}·rad^{-2}) for two dihedrals (C28-C29-C210-C211 and C1-C3-C2-O21).
Step6.5	2 fs	10 ns	Position harmonic restrain (2 kJ·mol^{-1}·Å^{-2}) for the backbone non-hydrogen atoms of protein and peptide;<br>Position restrain (0.5 kJ·mol^{-1}·Å^{-2}) for the sidechain non-hydrogen atoms of protein and peptide;<br>Planar harmonic restraint (0.4 kJ·mol^{-1}·Å^{-2}) for the phosphorus atom of POPC along the Z-axis;<br>Dihedral restraint (100 kJ·mol^{-1}·rad^{-2}) for two dihedrals (C28-C29-C210-C211 and C1-C3-C2-O21).
Step6.6	2 fs	10 ns	Position harmonic restrain (0.5 kJ·mol^{-1}·Å^{-2}) for the backbone non-hydrogen atoms of protein and peptide;
Step7	2 fs	1000 ns	Restrain-free

Supplementary References

1 Cherezov, V. *et al.* High-resolution crystal structure of an engineered human β_2-adrenergic G protein-coupled receptor. *Science* **318**, 1258-1265 (2007).

2 Zhang, X. *et al.* Structures of the human cholecystokinin receptors bound to agonists and antagonists. *Nat Chem Biol* **17**, 1230-1237 (2021).

3 Yin, Y. L. *et al.* Molecular basis for kinin selectivity and activation of the human bradykinin receptors. *Nat Struct Mol Biol* **28**, 755-761 (2021).

4 Zhu J. *et al.* Identification of the domains in RXFP4 (GPCR142) responsible for the high affinity binding and agonistic activity of INSL5 at RXFP4 compared to RXFP3 (GPCR135). *Eur J Pharmacol* **590**, 43-52 (2008).
