# Supplementary material for: Ligand recognition mechanism of the human relaxin family peptide receptor 4 (RXFP4)
Source: Nat Commun. 2023 Jan 30;14:492. doi: 10.1038/s41467-023-36182-z (PMC9886975; doi:10.1038/s41467-023-36182-z)
Supplement: Supplementary file 3 — Reporting Summary [file 41467_2023_36182_MOESM3_ESM.pdf]

Corresponding author(s): Ming-Wei Wang
Last updated by author(s): Jan 9, 2023

Reporting Summary

Nature Portfolio wishes to improve the reproducibility of the work that we publish. This form provides structure for consistency and transparency in reporting. For further information on Nature Portfolio policies, see our Editorial Policies and the Editorial Policy Checklist.

Statistics

For all statistical analyses, confirm that the following items are present in the figure legend, table legend, main text, or Methods section.

n/a	Confirmed	
☐	☒	The exact sample size (*n*) for each experimental group/condition, given as a discrete number and unit of measurement
☐	☒	A statement on whether measurements were taken from distinct samples or whether the same sample was measured repeatedly
☐	☒	The statistical test(s) used AND whether they are one- or two-sided<br>*Only common tests should be described solely by name; describe more complex techniques in the Methods section.*
☒	☐	A description of all covariates tested
☒	☐	A description of any assumptions or corrections, such as tests of normality and adjustment for multiple comparisons
☐	☒	A full description of the statistical parameters including central tendency (e.g. means) or other basic estimates (e.g. regression coefficient) AND variation (e.g. standard deviation) or associated estimates of uncertainty (e.g. confidence intervals)
☐	☒	For null hypothesis testing, the test statistic (e.g. *F*, *t*, *r*) with confidence intervals, effect sizes, degrees of freedom and *P* value noted<br>*Give P values as exact values whenever suitable.*
☐	☒	For Bayesian analysis, information on the choice of priors and Markov chain Monte Carlo settings
☒	☐	For hierarchical and complex designs, identification of the appropriate level for tests and full reporting of outcomes
☒	☐	Estimates of effect sizes (e.g. Cohen's *d*, Pearson's *r*), indicating how they were calculated

Our web collection on statistics for biologists contains articles on many of the points above.

Software and code

Policy information about availability of computer code

Data collection	Automated data collection on the Titan Krios was performed using serial EM 3.7.
Data analysis	The following software was used in the cryo-EM data processing, model building and structure validation: PyMOL v2.1, MotionCor2.1, Gctf v1.06, cryoSPARC v3.2.0, RELION-3.1.1, UCSF Chimera v1.13.1, UCSF ChimeraX 1.0, Phenix v1.18, Coot 0.9.4.1, Bsoft package 2.0.3 and DeepEmhancer 0.14. The molecular dynamic simulations were performed by Gromacs 2020.1, Schrodinger 2017-4, CHARMM-GUI v3.5, FreeSASA 2.0 and LINCS algorithm. The functional data were analyzed by GraphPad Prism 8.3. and NovoExpress 1.2.1. The MS analysis was conducted using the Qualbrowser application of Xcalibur software 2.1 and ProMass Deconvolution 2.8.

For manuscripts utilizing custom algorithms or software that are central to the research but not yet described in published literature, software must be made available to editors and reviewers. We strongly encourage code deposition in a community repository (e.g. GitHub). See the Nature Portfolio guidelines for submitting code & software for further information.

Data

Policy information about availability of data

All manuscripts must include a data availability statement. This statement should provide the following information, where applicable:

- Accession codes, unique identifiers, or web links for publicly available datasets
- A description of any restrictions on data availability
- For clinical datasets or third party data, please ensure that the statement adheres to our policy

All relevant data are available from the corresponding authors upon reasonable request. The raw data underlying Figs. 1e, 2f–g, 3c,3e,3h, 5b, Supplementary Figs. 1f–g, 2e-g, 3b-d, 4a-c, 10b-g and Supplementary Tables 3-4 were provided as a Source Data file. The atomic coordinates and electron microscopy maps have been deposited in the Protein Data Bank (PDB) under accession codes: 7YJ4 (INSL5–RXFP4–Gi complex) [http://dx.doi.org/10.2210/pdb7YJ4/pdb], 7YK6 (compound 4–RXFP4–Gi complex) [http://dx.doi.org/10.2210/pdb7YK6/pdb] and 7YK7 (DC591053–RXFP4–Gi complex) [http://dx.doi.org/10.2210/pdb7YK7/pdb] and Electron Microscopy Data Bank (EMDB) accession codes: EMD-33871 (INSL5–RXFP4–Gi complex) [https://www.ebi.ac.uk/pdbe/entry/emdb/EMD-33871], EMD-33888 (compound 4–RXFP4–Gi complex) [https://www.ebi.ac.uk/pdbe/entry/emdb/EMD-33888] and EMD-33889 (DC591053–RXFP4–Gi complex) [https://www.ebi.ac.uk/pdbe/entry/emdb/EMD-33888], respectively. The uncropped gels shown in Supplementary Figure 1c and Supplementary Figure 3b-d were displayed in Source Data.

Human research participants

Policy information about studies involving human research participants and Sex and Gender in Research.

Reporting on sex and gender	*Use the terms sex (biological attribute) and gender (shaped by social and cultural circumstances) carefully in order to avoid confusing both terms. Indicate if findings apply to only one sex or gender; describe whether sex and gender were considered in study design whether sex and/or gender was determined based on self-reporting or assigned and methods used. Provide in the source data disaggregated sex and gender data where this information has been collected, and consent has been obtained for sharing of individual-level data; provide overall numbers in this Reporting Summary. Please state if this information has not been collected. Report sex- and gender-based analyses where performed, justify reasons for lack of sex- and gender-based analysis.*
Population characteristics	*Describe the covariate-relevant population characteristics of the human research participants (e.g. age, genotypic information, past and current diagnosis and treatment categories). If you filled out the behavioural & social sciences study design questions and have nothing to add here, write "See above."*
Recruitment	*Describe how participants were recruited. Outline any potential self-selection bias or other biases that may be present and how these are likely to impact results.*
Ethics oversight	*Identify the organization(s) that approved the study protocol.*

Note that full information on the approval of the study protocol must also be provided in the manuscript.

Field-specific reporting

Please select the one below that is the best fit for your research. If you are not sure, read the appropriate sections before making your selection.

☒ Life sciences ☐ Behavioural & social sciences ☐ Ecological, evolutionary & environmental sciences

For a reference copy of the document with all sections, see nature.com/documents/nr-reporting-summary-flat.pdf

Life sciences study design

All studies must disclose on these points even when the disclosure is negative.

Sample size	No statistical methods were used to predetermine sample size. All functional data were obtained from at least three independent experiments to ensure each data point was repeatable. Sample size for the cryo-EM studies was determined by availability of microscope time and to ensure unambiguous modeling of the structures.
Data exclusions	No data were excluded.
Replication	Experimental findings were reliably reproduced at least three independent times.<br>All functional assays were performed in technical quadruplicate or triplicate. All attempts at replication were successful.
Randomization	Randomization was not required.<br><br>Randomization is not relevant to this study, as protein samples are not required to be allocated into experimental groups in structural studies, and no animals or human research participants are involved in this study.
Blinding	Blinding was not performed.

Blinding

Blinding is not relevant to this study, as protein samples are not required to be allocated into experimental groups in protein structural studies, and no animals or human research participants are involved in this study.

Reporting for specific materials, systems and methods

We require information from authors about some types of materials, experimental systems and methods used in many studies. Here, indicate whether each material, system or method listed is relevant to your study. If you are not sure if a list item applies to your research, read the appropriate section before selecting a response.

Materials & experimental systems

n/a	Involved in the study
☐	☒ Antibodies
☐	☒ Eukaryotic cell lines
☒	☐ Palaeontology and archaeology
☒	☐ Animals and other organisms
☒	☐ Clinical data
☒	☐ Dual use research of concern

Methods

n/a	Involved in the study
☒	☐ ChIP-seq
☐	☒ Flow cytometry
☒	☐ MRI-based neuroimaging

Antibodies

Antibodies used

Anti-Flag primary antibody (mouse monoclonal), clone M2, purified immunoglobulin (Purified IgG1 subclass), Sigma-Aldrich, Cat#F3165; Anti-mouse Alexa Fluor 488 conjugated secondary antibody (Donkey Polyclonal), Invitrogen, Cat#A-21202

Validation

Anti-Flag primary antibody (mouse monoclonal), applies to western Blot, immunohistochemis, ELISA, flow cytometry and immunoprecipitatin; Anti-mouse Alexa Fluor 488 conjugated secondary antibody (Donkey Polyclonal).

Eukaryotic cell lines

Policy information about cell lines and Sex and Gender in Research

Cell line source(s)

Sf9 (Invitrogen, DOI: 10.1126/science.aav7942)
High-Five insect cells (Thermo Fisher Scientific,Cat#B85502)
HEK293T (ATCC Cat#64127316)
CHO-K1 (ATCC Cat#CCL-61)

Authentication

No authentication required.

Mycoplasma contamination

Cell lines were tested and free from mycoplasma contamination.

Commonly misidentified lines
(See ICLAC register)

No commonly misidentified cell lines were used.

Flow Cytometry

Plots

Confirm that:

☒ The axis labels state the marker and fluorochrome used (e.g. CD4-FITC).

☒ The axis scales are clearly visible. Include numbers along axes only for bottom left plot of group (a 'group' is an analysis of identical markers).

☒ All plots are contour plots with outliers or pseudocolor plots.

☒ A numerical value for number of cells or percentage (with statistics) is provided.

Methodology

Sample preparation

Sample preparation listed in Methods.

Instrument

Flow Cytometer, BD Accuri C6

Software

NovoExpress 1.2.1

Cell population abundance

Approximately 20,000 cellular events were collected and the total fluorescence intensity of positive expression cell population was calculated.

Gating strategy

Gating was determined by the Alexa-488 fluorescence intensity to differentiate positive cells and all other cells.

☒ Tick this box to confirm that a figure exemplifying the gating strategy is provided in the Supplementary Information.
